# Genome-wide identification of bHLH transcription factors and their response to salt stress in *Cyclocarya paliurus*

**DOI:** 10.3389/fpls.2023.1117246

**Published:** 2023-03-09

**Authors:** Zijie Zhang, Jie Fang, Lei Zhang, Huiyin Jin, Shengzuo Fang

**Affiliations:** ^1^ College of Forestry, Nanjing Forestry University, Nanjing, China; ^2^ Co-Innovation Center for Sustainable Forestry in Southern China, Nanjing, China

**Keywords:** wheel wingnut, *bHLH* family genes, *CpbHLH* genes, salt tolerance, expression analysis, regulation networks

## Abstract

As a highly valued and multiple function tree species, the leaves of *Cyclocarya paliurus* are enriched in diverse bioactive substances with healthy function. To meet the requirement for its leaf production and medical use, the land with salt stress would be a potential resource for developing *C. paliurus* plantations due to the limitation of land resources in China. The basic helix-loop-helix (bHLH) transcription factor protein family, the second largest protein family in plants, has been found to play essential roles in the response to multiple abiotic stresses, especially salt stress. However, the *bHLH* gene family in *C.paliurus* has not been investigated. In this study, 159 *CpbHLH* genes were successfully identified from the whole-genome sequence data, and were classified into 26 subfamilies. Meanwhile, the 159 members were also analyzed from the aspects of protein sequences alignment, evolution, motif prediction, promoter cis-acting elements analysis and DNA binding ability. Based on transcriptome profiling under a hydroponic experiment with four salt concentrations (0%, 0.15%, 0.3%, and 0.45% NaCl), 9 significantly up- or down-regulated genes were screened, while 3 genes associated with salt response were selected in term of the GO annotation results. Totally 12 candidate genes were selected in response to salt stress. Moreover, based on expression analysis of the 12 candidate genes sampled from a pot experiment with three salt concentrations (0%, 0.2% and 0.4% NaCl), *CpbHLH36/68/146* were further verified to be involved in the regulation of salt tolerance genes, which is also confirmed by protein interaction network analysis. This study was the first analysis of the transcription factor family at the genome-wide level of *C. paliurus*, and our findings would not only provide insight into the function of the *CpbHLH* gene family members involved in salt stress but also drive progress in genetic improvement for the salt tolerance of *C. paliurus*.

## 1 Introduction

Plants are constantly challenged by the environmental stresses, and it is estimated that up to 70% of plants can be affected by diverse abiotic stresses from which they cannot escape (Mantri et al., 2012a; Chen et al., 2021). Salinity stress, which affects 8.31 billion hm^2^ of land, is one of the major abiotic stresses to impair plant growth (Li et al., 2020a; Zhang et al., 2020a; Liao et al., 2022). In order to maintain normal growth and survival, plants turned on or suppressed many genes by transcription factors (TFs) to regulate physiological and biochemical processes in response to changes in the external environment (Agarwal et al., 2006; Bhatnagar-Mathur et al., 2008). It has been noted that six major families of transcription factors (TFs) have vital regulatory functions in plant resistance to various abiotic stresses, including MYBs, basic helix-loop-helix (bHLHs), ethylene responsive element binding factor (ERFs), dehydration responsive element-binding (DREBs), WRKYs and basic region/leucine zipper motif members (bZIPs) (Kavas et al., 2016; Mao et al., 2017; Zhang et al., 2022a). As reported, the bHLH TFs are widespread in all eukaryotes and own the second largest number of TF families in plants (Pires and Dolan, 2010; Feller et al., 2011), while the bHLH members possess highly conserved bHLH domain constituted by two functionally diverse regions with approximately 60 amino acids (Toledo-Ortiz et al., 2003). The basic region, containing approximately 10-17 amino acids and a binding site to bind the specific E-box (CANNTG) DNA sequence, is located at the N-terminus. Inversely, at the C-terminus, the helix-loop-helix (HLH) region, consisting of roughly 40 amino acids and acting as a dimerization domain, is responsible for facilitating the dimerization between proteins (Atchley et al., 1999). On account of diverse binding elements, bHLH transcription factors in animals were organized into six groups (Wang et al., 2018b), whereas the classification of plant bHLH proteins has not been determined though 15-32 groups were suggested according to current studies (Pires and Dolan, 2010).

Over the years, many plant bHLH proteins have been identified and characterized. For example, there are 162 *bHLH* genes in *Arabidopsis thaliana* (Toledo-Ortiz et al., 2003), 188 in apple (*Malus* × *domestica*) (Mao et al., 2017), and 113 in strawberry (*Fragaria* × *ananassa*) (Zhao et al., 2018), 115 in spine grapes (*Vitis davidii*)(Li et al., 2021) and 206 in sweet osmanthus (*Osmanthus fragrans*) (Li et al., 2020b). Furthermore, some studies on the role of bHLH proteins revealed that the plant bHLH family participated in numerous processes including anthocyanin biosynthesis (Hou et al., 2017; Lim et al., 2017), growth and development (Sorensen et al., 2003; Carretero-Paulet et al., 2010) and response to stress (Babitha et al., 2013; Ji et al., 2016; Sum et al., 2021). Among the functions, regulating the stress tolerance by binding to the promoters of downstream genes has been well characterized in bHLH proteins (Cui et al., 2016). For instance, *MdbHLH104* was recognized to response to iron deficiency stress in apple by immediately binding to the P3 cis-acting element of the *MdAHA8* promoter (Zhao et al., 2016), while *ICE1 (AtbHLH116*), could increase the cold tolerance in *A. thaliana* by activating expression the cold-responsive (COR) genes (Chinnusamy et al., 2003). Besides, gene *AtbHLH92* has been shown to have function in responses to osmotic stresses of plants (Jiang et al., 2009) and AtbHLH17 (AtAIB), a nuclear-localized bHLH-type protein, could confer the drought tolerance of transgenic plants *via* regulating of ABA signaling (Li et al., 2007). More interesting is some bHLH TFs could play essential role in the regulation of multiple abiotic stresses signaling simultaneously. For instance, *SlICE1a* (a tomato bHLH transcription factor) could enhance the resistance of cold, osmotic and salt stresses (Feng et al., 2013), while overexpressed *TabHLH39* in *A. thaliana* could increase freezing, salt, and drought tolerance (Zhai et al., 2016). It was also reported that ATNIG1 regulates downstream gene expression by specifically binding to E-box motifs (CANNTG) of salt stress-related gene promoters, thereby enhanced plant tolerance to salt stress (Kim and Kim, 2006).

Wheel wingnut (*Cyclocarya paliurus*), a multiple-fuction tree species, belongs to Juglandaceae family (Fang, 2022). Although now naturally distributed in sub-tropical mountain areas of China, *Cyclocarya* has a long fossil record of fruits in North America, Europe and eastern Asia, while went extinct in North America and Europe during the Cenozoic (Manchester et al., 2009; Wu et al., 2017). The leaves of *C. paliurus* has been used as tea, traditional food and medicine for thousands of years in China (Fang et al., 2006), and the leaves have been listed as new food raw material by National Health and Family Planning Commission of China since 2013 (Qin et al., 2021). Many studies have demonstrated that the extractives from *C. paliurus* leaves possess antioxidant activities, antiproliferative activities and antidiabetic activities (Kurihara et al., 2003; Yao et al., 2015; Zhai et al., 2018; Zhou et al., 2021), and some products derived from the leaves have been developed and put into the market. However, at present, the resources of *C. paliurus* are mainly distributed in natural forests whereas its plantations can only be established at the sites where the soil is relatively deep and loose, well-drained and moist fertile (Fang et al., 2011; Fang, 2022), resulting in that the amount of its leaves cannot meet the market demand (Qin et al., 2021). Therefore, a feasible option is to develop *C. paliurus* plantation with oriented cultivation on potential land resources such as coastal saline areas due to the limitation of land resources in China in order to meet the requirement for its leaf production and medical use. Our previous studies found that R2R3-MYB transcription factor family affected salt tolerance of *C. paliurus* (Zhang et al., 2022b), while some bHLH proteins could regulate the accumulation of flavonoid compounds under salt stress by promoting the expression of genes encoding related enzymes in *C. paliurus* (Zhang et al., 2021), which provide some evidences for the crucial role of TFs in plant resistance to salt stress. However, so far, no TF family has been systematically identified in the whole genome of *C. paliurus*. The recent release of high quality whole-genome sequence data of *C. paliurus* gives us the opportunity to investigate the *bHLH* gene family and to identify salt-responsive members. In this study, 159 bHLH transcription factors in *C. paliurus* were analyzed comprehensively and systematically, and some key *bHLH* genes associated with salt tolerance were identified. Results from this study would not only provide insight into the function of the *CpbHLH* gene family members involved in salt stress, but also drive progress in genetic improvement for the salt tolerance of *C. paliurus* to develop *C. paliurus* plantation in the coastal saline areas of south-east China.

## 2 Materials and methods

### 2.1 Identification and sequence analysis of *CpbHLH* genes

The whole genome data of *C. paliurus* were available from the Genome Sequence Archive (GSA) database (https://ngdc.cncb.ac.cn/gsa) provided by our research group. The Hidden Markov Model (HMM) profile of the HLH domain (PF00010) was obtained from the Pfam database (version 30.0) (Finn et al., 2014), and was used as a query to search for all protein sequences with default E-values in the whole genome and to identify genes with specific conserved domains by HMMER software (version 3.3; http://hmmer.org/) (Johnson et al., 2010). All screened sequences were aligned and checked with the online tools Batch CD-search (https://www.ncbi.nlm.nih.gov/Structure/bwrpsb/bwrpsb.cgi) (Marchler-Bauer and Bryant, 2004), Pfam, and SMART (http://smart.embl-heidelberg.de) (Letunic et al., 2021) to verify the existence of the conserved bHLH domain. The ExPASy software (https://web.expasy.org/protparam/) was used to obtain basic physical and chemical characteristics of these *bHLH* genes respectively.

### 2.2 Phylogenetic analysis, multiple alignment analysis and chromosomal locations

The *A. thaliana* MYB sequences data were download from PlantTFDB database (http://planttfdb.cbi.pku.edu.cn/index). The construction of a phylogenetic tree consisted of proteins from *A. thaliana* and *C. paliurus* was performed with MEGA X (version 6.0) (Kumar et al., 2018) software using the neighbor-joining (NJ) method with 1000 bootstrap replicates. Multiple sequence alignment (MSA) of *C. paliurus* and *A. thaliana* bHLH proteins was performed using ClustalX 2.11 software (Thompson et al., 1997), and Weblogo3 (http://weblogo.threeplusone.com/create.cgi), while Jalview software (http://www.jalview.org/) was used to visualize and analyze the sequences of conserved domains in CpbHLH proteins. The GFF3 (Generic Feature Format Version 3) file, containing the positional and gene structure information of genes on the chromosomes, was obtained from whole genome data of *C. paliurus*. The TBtools software (version 1.098774) (Chen et al., 2020) was adopted to map the *CpbHLH* genes onto specific chromosomes.

### 2.3 Gene structure, conserved motif, and promoter analysis

The exon/intron structures of *CpbHLH* genes were visualized by TBtools software (version 1.098774) (Chen et al., 2018), whereas fifteen conserved motifs were obtained using the online software MEME (http://MEME-suite.org/) (upper limit of the recognition motif was 20, minimum motif width was 6, and maximum motif width was 50, zoops) (Bailey et al., 2009). The online tool PLACE (Higo et al., 1999) was used to analyze the cis-acting elements of *CpbHLH* genes.

### 2.4 RNA-seq data analysis, GO annotation and prediction of the protein interaction network

Raw data were obtained *via* RNA sequencing of leaves treated with different salt concentration in hydroponic experiment (Zhang et al., 2021). *CpbHLHs* with reads per kilobase of transcript per million mapped reads or fragments per kilobase of transcript per million mapped reads (RPKM and FPKM, respectively) > 1 were collected for further analyses of all of the transcriptome data. TBtools was performed to generate the heatmap (Chen et al., 2020). Gene ontology (GO) analysis was carried out by the Blast2GO program (Conesa et al., 2005), with selecting the NCBI database as the reference database. The results were divided into three categories, namely molecular function, biological process, and cellular component. The NCBI database (https://www.ncbi.nlm.nih.gov/) were used to search the functions of *AtbHLHs*, which were predicted to be orthologous genes of *CpbHLHs*. STRING (https://string-db.org/) (Szklarczyk et al., 2019) was performed to predict the functional interaction network of candidate genes with option value>0.7.

### 2.5 Plant materials and stress treatments

The experiment was carried out at Baima Experimental Base of Nanjing Forestry University (31°35′ N, 119°09′ E). *C. paliurus* seeds were collected from Jinzhongshan county (24° 58′ N latitude, 110° 09′ E longitude), Guangxi province, China, in October 2018. After treated by exogenous GA3 (gibberellin A3) and stratification method (Fang et al., 2006), the germinated seeds were sown in nonwoven containers (10.0 cm height, 8.0 cm diameter) in April 2019.

**Hydroponic experiment:** After three months, uniform size seedlings (height: 40 ± 2.79 cm) were selected and transplanted to polypropylene containers (50L) with 1/2-strength Hoagland’s nutrient solution (pH 6.0 ± 0.2). Two weeks after hydroponic transplanting, four salt concentration (0%, 0.15%, 0.3%, and 0.45% NaCl) regimes were implemented in completely randomized design with three biological replicates for each treatment. The detailed information has been described in our previous study (Zhang et al., 2022a).

**Pot experiment:** After one-year growth in the nonwoven containers, the seedlings were transplanted into the big nonwoven containers (25 cm height, 20 cm diameter) and cut into 3-5 cm height in early spring in 2020. In February 2022, saplings with similar size were selected and all their stems were cut to 120 cm height, whereas in early April 2022, the selected saplings were transplanted from the nonwoven containers into plastic pots (26 cm height, 26 cm top diameter and 20 cm bottom diameter) containing peat: substrates of perlite: rotten bird dung: soil =5: 2:2:1 (v/v/v/v). The plastic pots were placed in plastic trays to prevent NaCl leaching. The substrate was a loam with pH 6.4, and the contents of total N, total P, and total K in the soil were 79.7, 66.5, 2.40, and 9.7 g kg^−1^, respectively.

**Figure 1:**
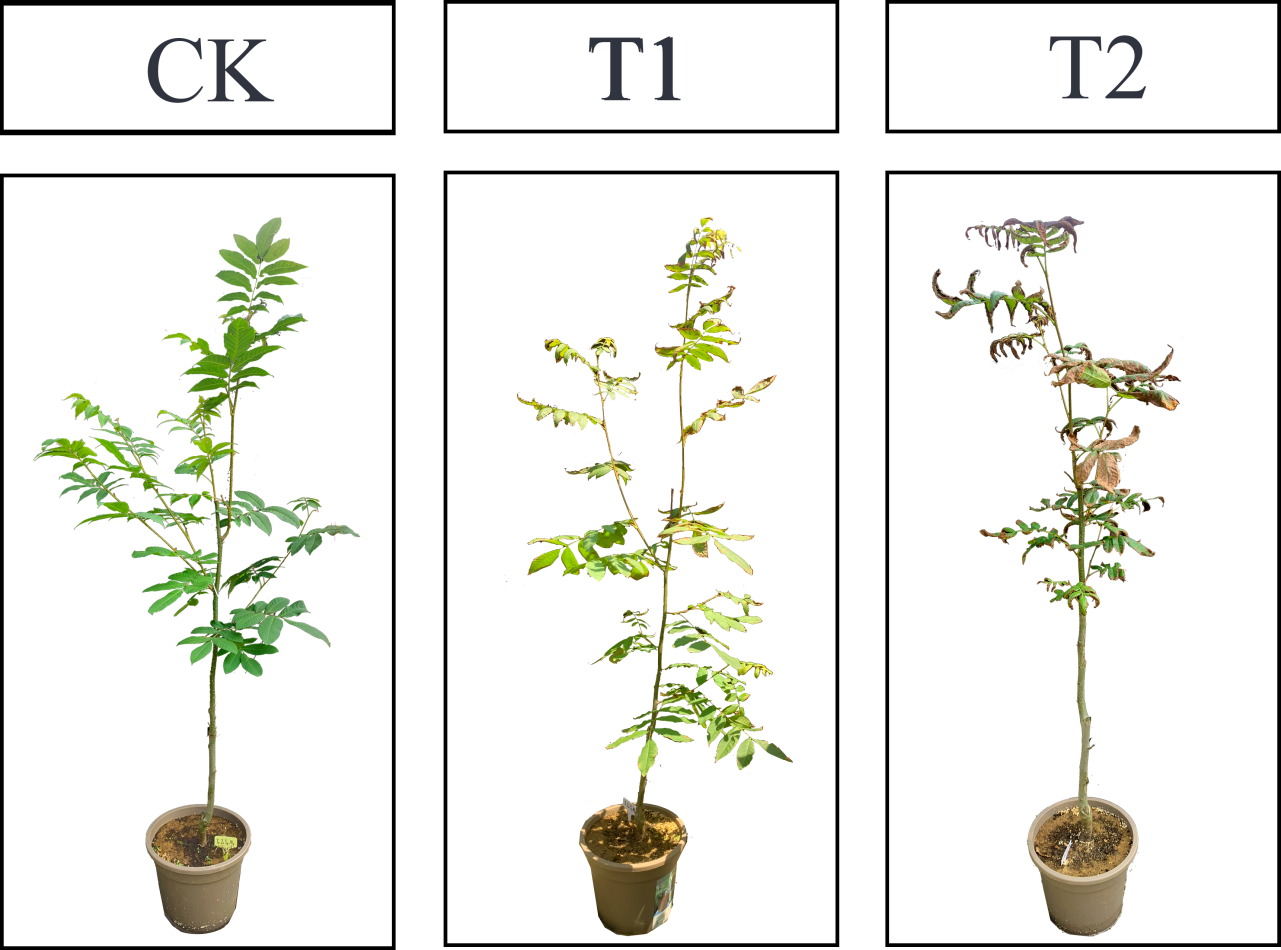
Phenotypes of *C. paliurus* seedlings at the sampling time under various salt treatments of the pot experiment.

Salt treatments were conducted in early May 2022, and a completely randomized design was adopted with three replications per treatment and six plants per replication. Based on previous research (Zhang et al., 2022b), three levels of NaCl concentration were set up: CK (control, distilled water), T1 (0.2% NaCl) and T2 (0.4% NaCl). 1L solution were gradually add to the soil every three days (Chen et al., 2021), and electrical conductivity in the substrate was also monitored to keep the soil salt concentration relatively stable. Six complete and mature leaves were respectively collected from the upper, middle and lower positions of each sampled tree at the 45 days after the treatments (obvious differences were observed) (**Figure 1**) and were immediately frozen in liquid nitrogen and stored at −80°C until needed for further analysis.

### 2.6 RNA extraction and real-time quantitative RT-PCR analysis

Plant materials were ground under RNase-free conditions. Trizol reagent kit (Invitrogen, Carlsbad, CA, USA) was used to extract RNA from 9 samples of the 3 treatments (CK, 0.2% NaCl and 0.4% NaCl); subsequently, MonScript RTIII All-in-One Mix with dsDNase kits (Monad, Nanjing, China) was used to acquire cDNA, following the manufacturer’s instructions. The qRTPCRs were performed on BiosystemsTM 7500 Real-Time PCR Systems (Monad, China). Primer Premier 6.0 (Premier Biosoft International, Palo Alto CA, USA) was used to design qRT-PCR primers for 12 genes (**Supplementary Table S1**). SYBR Premix Ex Taq kit (Takara Biotechnology, Dalian, China) was applied to conduct qRT-PCR analysis. The cDNA diluted 20 times and an *18sRNA* gene (Chen et al., 2019) were selected as the template and the internal standard, respectively. PCR reaction conditions were as 95 °C for 3 min; denaturation 5 s at 95 °C; 60 °C for 30 s; 40 cycles. Three technical and three biological replicates were used for each sample. After reaction, the relative expression levels of target gene and internal reference gene were calculated with the 2^−ΔΔCT^ method (Penfield, 2001).

### 2.7 Statistical analysis

One-way analysis of variance (ANOVA) was conducted to identify significant differences in the related gene expression among the treatments, followed by Duncan’s test for multiple comparisons. All statistical analyses were performed using IBM SPSS Statistics Version 22 software package (SPSS Inc., IBM Company Headquarters, Chicago, IL, USA). Data were presented as means ± standard deviation (SD).

## 3 Results

### 3.1 Identification and sequence analysis of *CpbHLH* genes

**Figure 2:**
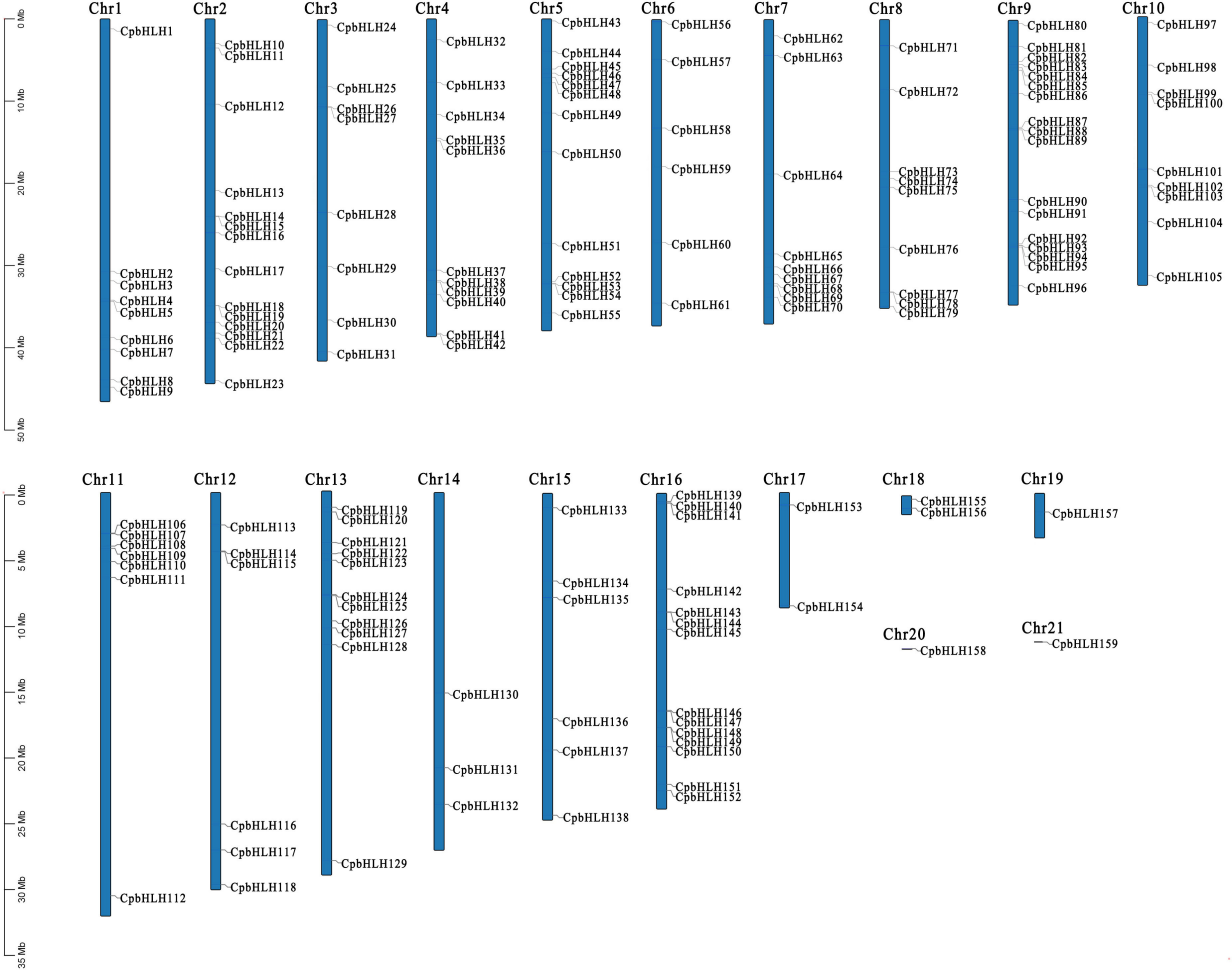
Chromosomal locations of the *CpbHLH* genes. The 159 *CpbHLH* genes were distributed on 21 pseudo-chromosomes of *C. paliurus* based on their physical positions.

Based on the Genome-wide data of *C. paliurus*, a total of 174 supposed CpbHLH proteins were discovered by using the HMMER software with default parameters. Subsequently, SMART and CD-Search were performed to confirm the existence of the conserved bHLH domain. After removing redundant sequences, 159 bHLH protein sequences of *C. paliurus* with typical complete bHLH domain were obtained and they were named *CpbHLH1* to *CpbHLH159* according to their location on chromosomes (**Figure 2**; **Supplementary Table S2**). Sequence analysis showed that the average length of the CpbHLH proteins was 354 amino acids. The relative molecular weight (Nw) ranged from 10454.72 Da (*CpbHLH39*) to 175494.7 Da (*CpbHLH86*), whereas the isoelectric point (pI) ranged from 4.65 (*CpbHLH48*) to 9.66 (*CpbHLH66*) (**Supplementary Table S2**).

### 3.2 Conserved residues and DNA-binding ability prediction of the *CpbHLH* genes

**Figure 3:**
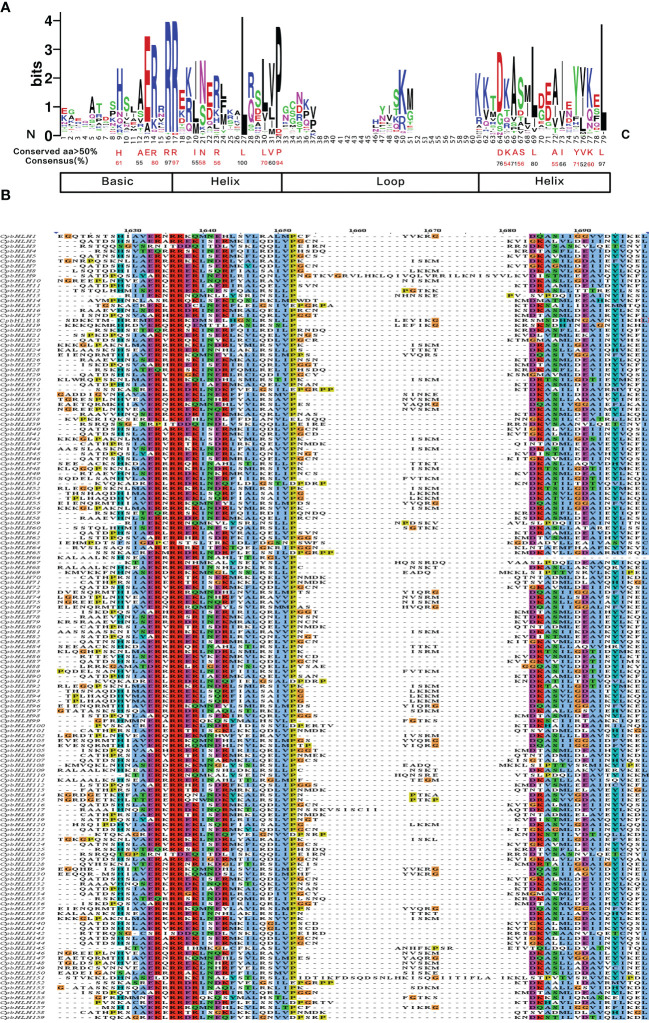
Multiple sequence alignments of the bHLH domains in CpbHLH proteins. **(A)** Visualization of conserved amino acids of bHLH domains of CpbHLH proteins. Amino acids with a conserved degree of more than 50 and their conserved degree were labeled using red and black colors for easy recognition which had no special meaning. **(B)** Multiple sequence alignments of the bHLH domains of 159 CpbHLH proteins, using the Clustal color scheme.

To gain in-depth knowledge of the function of CpbHLH family, the bHLH domains of the CpbHLH proteins were searched and the presence of the conserved amino acid residues were analyzed based on multiple sequence alignment. The alignment results (**Figure 3**) showed that the CpbHLH domains were composed of four conserved regions, namely one basic region, two helix regions and a loop region. Consistent with previous studies (Heim et al., 2003), the conservation of basic region and helix region is higher than that of the loop region. The bHLH domains of *C. paliurus* were made up of 79 amino acid residues, of which 24 were highly conserved (> 50% consensus ratio) and 8 were extremely conservative (> 75% consensus ratio). Among the 24 highly conserved amino acid residues, six conserved residues were found in the basic region (His-9, Ala-12, Glu-13, Arg-14, Arg-16, Arg-17), seven conserved residues were found in the first helix region (Ile-20, Asn-21, Arg-23, Leu-27, Leu-30, Val-31, Pro-32), one conserved residues were found in the loop region (Asp-64), and ten conserved residues were found in the second helix region (Lys-65, Ala-66, Ser-67, Leu-69, Ala-72, Ile-73, Tyr-75, Val-76, Lys-77, Leu-79).

It is generally believed that the basic region performs DNA binding functions, and is critical for the bHLH family to achieve its biological function (Carretero-Paulet et al., 2010). Therefore, the DNA-binding ability of the 159 CpbHLH proteins were predicted based on the conserved amino acid residues in the basic region (**Supplementary Table S3**). The remaining 159 CpbHLH members were classified into three categories: G-box (His/Lys-9, Glu-13 and Arg-17), E-box (Glu-13 and Arg-16) and non-E-box (Glu-13 and Arg-16 do not appear together) in accordance with the classification method reported previously (Katiyar et al., 2012). The predicted results revealed there were 93 G-box-binding proteins, 43 non-G-box-binding proteins and 23 non-E-box-binding proteins in 159 CpbHLHs (**Supplementary Table S3**).

### 3.3 Phylogenetic analysis and classification of the *CpbHLH* genes

**Figure 4:**
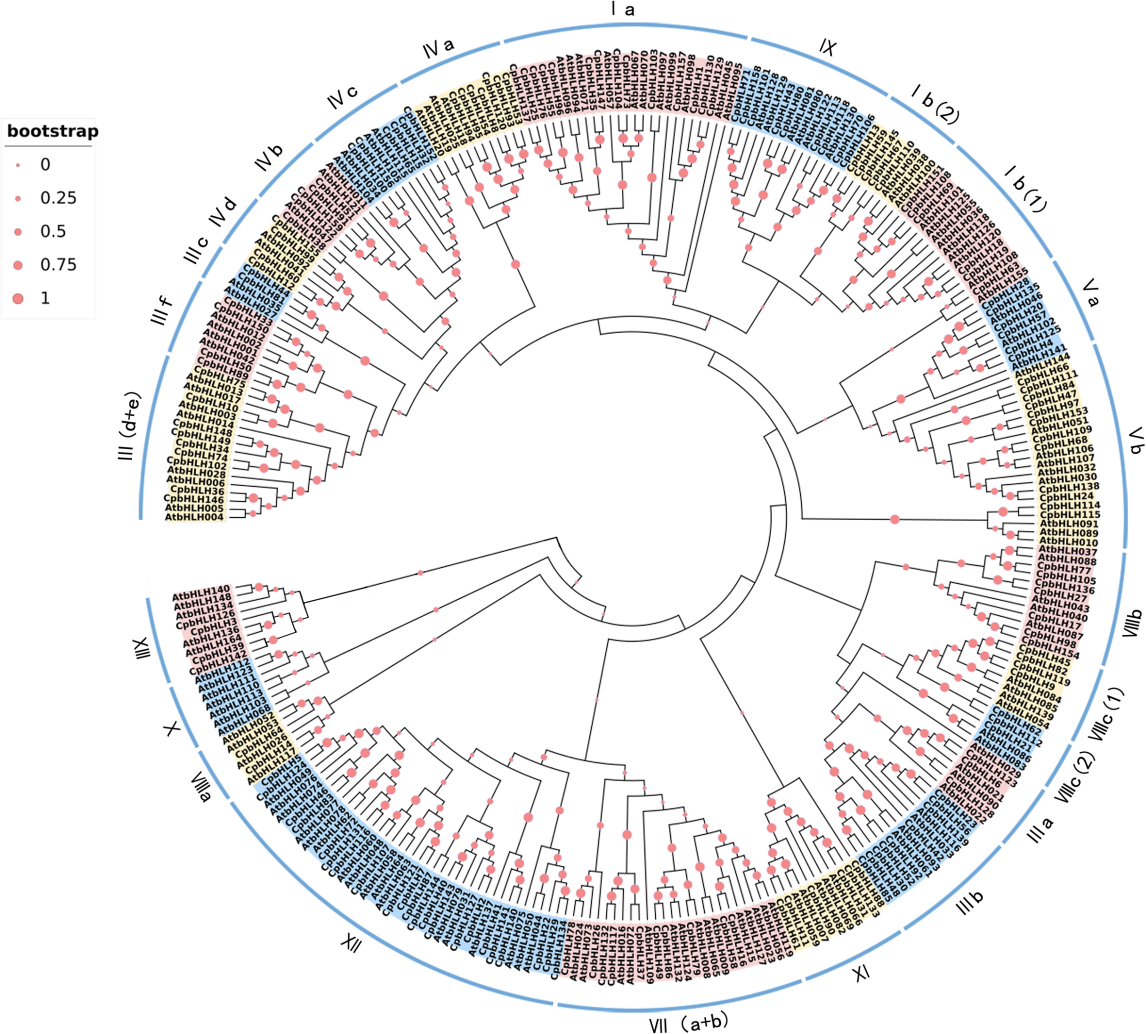
Phylogenetic tree and classification of bHLH subfamily proteins in *A. thaliana* and *C. paliurus*. The number of bHLH proteins of *A. thaliana* and *C. paliurus* is 140 and 159, respectively. The red dots represent boot values—the larger the dot, the larger the bootstrap value. Roman numerals line up with the bHLH subfamily.

In order to explore the evolutionary relationship among the CpbHLH members, the 159 CpbHLH proteins were aligned with 140 bHLH proteins from Arabidopsis, afterwards the phylogenetic tree was constructed using total 299 bHLH proteins based on the alignment (**Figure 4**). In accordance with the classification of bHLH proteins from Arabidopsis and other plants (Heim et al., 2003; Li et al., 2020b; Li et al., 2021), 299 bHLH protein sequences were classified into 26 subfamilies, and were named from Ia to XV on the basis of the nomenclature of *AtbHLHs* proposed by Heim et al. (Heim et al., 2003). **Figure 4** showed that the XII subfamily was the largest (contained 35 CpbHLH proteins), while the smallest subfamily (VI) contained only one CpbHLH protein. According to results from Heim et al. (Heim et al., 2003), CpbHLH proteins in the same subfamily would have similar functions, consequently, the clustering results of phylogenetic tree could contribute to predict the function of CpbHLH proteins.

### 3.4 Gene structure and conserved motif analysis of *CpbHLH* genes

**Figure 5:**
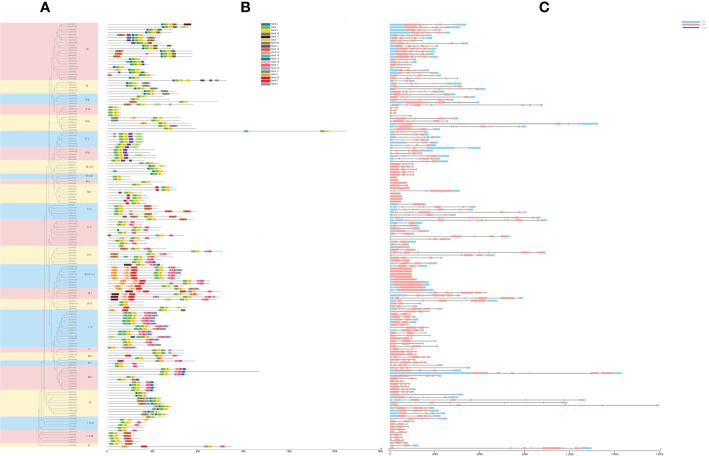
Analysis of conserved motifs and gene structure for 159 CpbHLH proteins. **(A)** Phylogenetic tree. **(B)** Distribution of conserved motifs. Twenty motifs were represented by twenty kinds of colored blocks. The position of each block represents the location of the motif. **(C)** organization of gene structure. The length of the gray line represents the length of a sequence relative to that of all the other sequences.

Diversity of exon-intron structures, which could cause divergences in coding regions, is significant to the evolution of multiple gene families (Xu et al., 2012b). Hence, the gene structural characteristics of the *CpbHLH* family were investigated. The number of exons in the 159 *CpbHLH* genes varied from 1 to 13 (**Figure 5C**). In addition, 20 (12.6%) genes were intronless and distributed across subfamilies IIId, IIIe, VIIIa, VIIIb and VIIIc(2), while 13 (8.2%) genes contained one intron, and certainly the remaining genes had two or more introns. The 159 genes in different families varied widely in structure, including the number and relative location of introns and exons (**Figure 5C**). On the contrary, the intron/exon patterns of genes in the same subfamily had highly similarity, such as in subfamilies Ib(1) (five three-exon genes), Ib(2) (five three-exon genes), III(d+e) (nine one-exon genes), IVc (eight five-exon genes), and VIIIb (seven one-exon genes) (**Figure 5C**).

It is generally accepted that motifs figure prominently in interaction and signal transduction between different modules of the gene transcription process (Toledo-Ortiz et al., 2003). To further understand the evolutionary relationships among these CpbHLH proteins, the conserved motifs were analyzed by using MEME. Twenty motifs were identified and their sequences and length were counted (**Figure 5B**, **Supplementary Table S4**). In addition, eight of twenty motifs were annotated by Pfam and CD-search (**Supplementary Table S4**). Obviously, the composition patterns tended to be consistent with the results from our phylogenetic tree and gene structures, being resemble among genes within the same group, but varying greatly between groups (**Figure 5**). The number of motifs in 159 *CpbHLHs* ranged from one (*CpbHLH66*) to nine (*CpbHLH50*). All 159 *CpbHLH* genes contained motif 1 and motif 2, except *CpbHLH66*, only containing motif 1 (**Figure 5B**). Interestingly, some conserved motifs were nested in specific groups. For example, motif 13 only existed in group Ia, motif 16 in group VIIIb, motif 18 in group XII, and motif 19 in group IX respectively (**Figure 5B**). This phenomenon might be the reason why functions for CpbHLH proteins tend to be specific to a particular group.

### 3.5 GO annotation and cis-element analyses of the *CpbHLHs*

**Figure 6:**
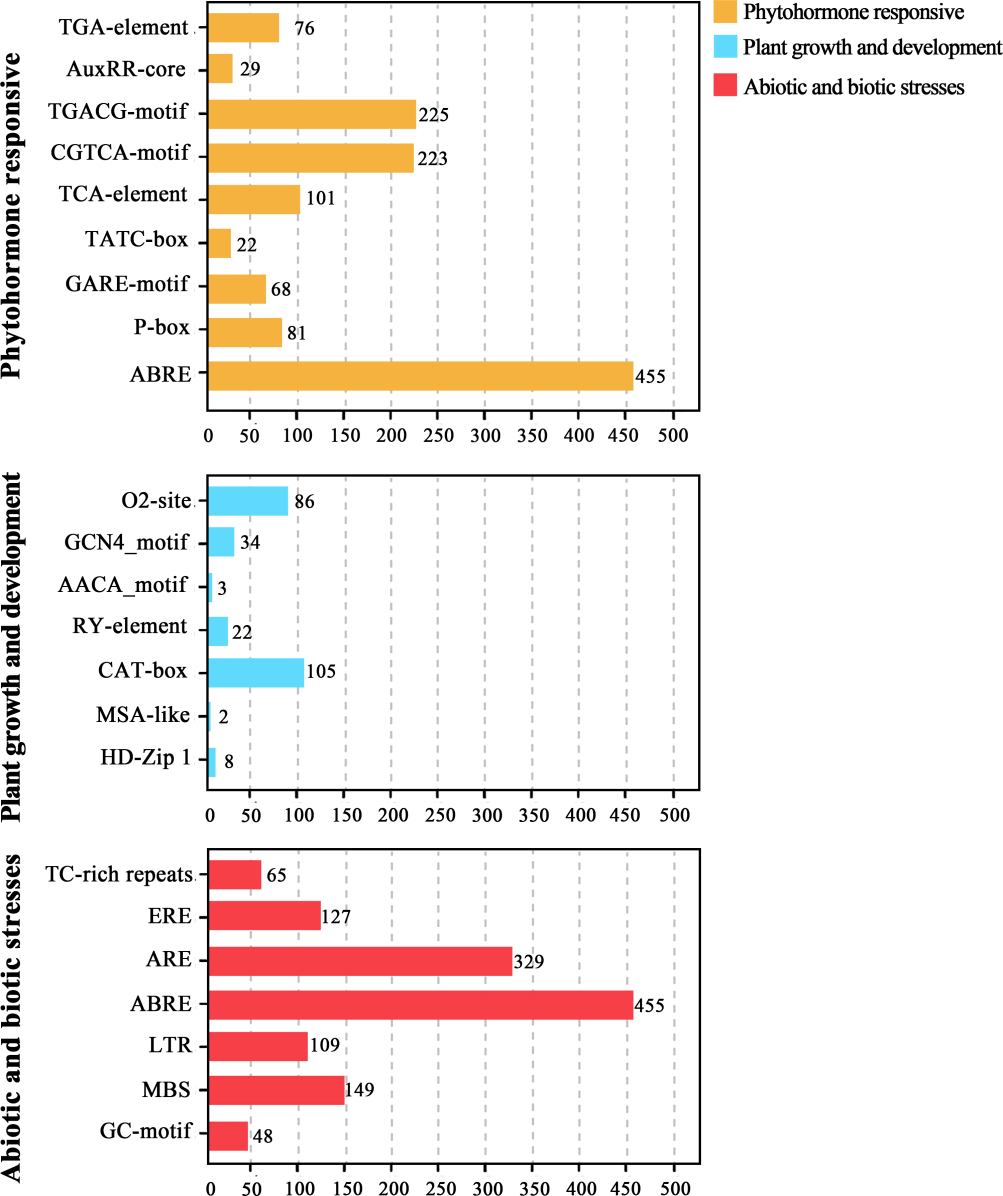
Gene ontology (GO) distribution of CpbHLH proteins. GO annotation using a cut-off value of *p* ≤ 0.05 showed that GO items including molecular function (MF), biological process (BP), and cellular component (CC), while predominant GO items was selected to visualize the result.

The highly differentiated sequences outside the conserved bHLH domain suggest that CpbHLH proteins may have a variety of biological functions. GO annotation of these 159 proteins was performed to understand the biological processes associated with *CpbHLH* genes. The results are shown in (**Figure 6**; **Supplementary Table S5**). The identified CpbHLH proteins were classified into three main Gene ontology (GO) terms, which were CC (cellular component), MF (molecular function), and BP (biological process). Within MF category, the majority of CpbHLH proteins were annotated for “molecular function” (139/159), “nucleic acid binding” and “DNA binding”, respectively. These functions were closely related to the primary roles that TFs have. As for CC category, most of the CpbHLH proteins were assigned to cellular components and the nucleus (139/159). However, there were also a small number of CpbHLH proteins distributed in cytoplasm (8/159), organelle part (7/159), cytosol (4/159), symplast (*CpbHLH37/117/132*) and chloroplast (*CpbHLH68/109*) (**Figure 6**; **Supplementary Table S5**). Furthermore, the BP aspect showed that CpbHLH proteins participated in various biological processes. Proteins annotated to be related to multiple biosynthetic and metabolic possessed the largest number of *CpbHLHs* (141/159). Besides, CpbHLH proteins may function in regulating biological processed, such as regulation of cellular process (111/159), transcription (109/159), DNA-templated (109/159) and gene expression (109/159). The BP analysis also showed that many *CpbHLHs* could respond to stimuli (46/159), including different types of biotic and abiotic stressors, while *CpbHLH38/68/109* were predicted to be involved in respond to salt stress (**Figure 6**; **Supplementary Table S5**).

**Figure 7:**
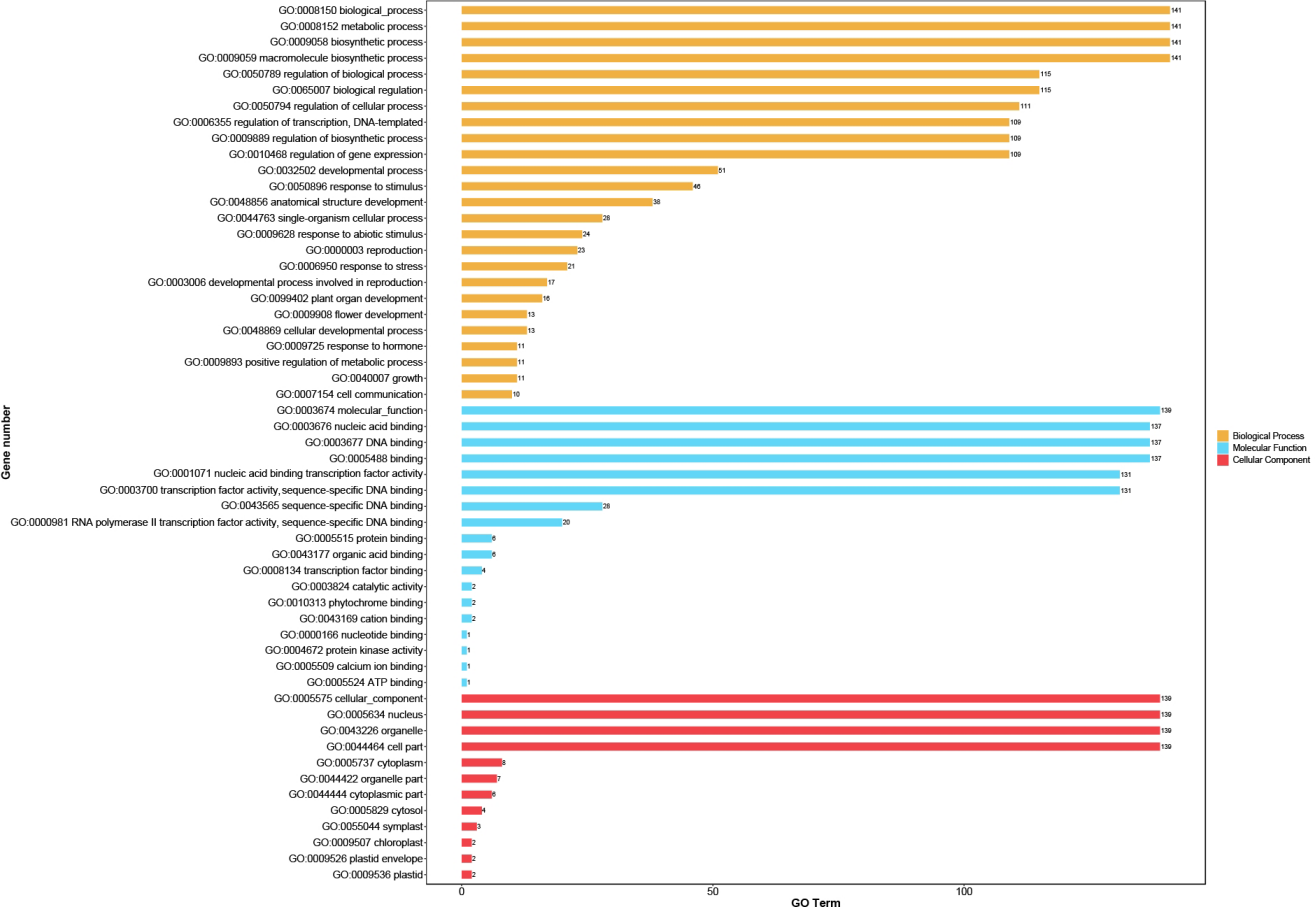
Cis-regulatory elements in the promoter region of *CpbHLH* genes. The figure represents the number of each type of motifs identified in the promoter sequence of *CpbHLH* genes.

Conserved motifs located in gene promoter regions are recognition and binding sites for proteins. In this study, a large number of cis-regulatory elements (CREs) of *CpbHLH* genes were identified, and they were classified into three main categories (plant growth and development, phytohormone responsive, as well as abiotic and biotic stresses) according to their roles (**Figure 7**). Our result showed that CAT-box (105) and O2-site (86), which were involved in the meristem expression and zein metabolism regulation respectively were most frequently found motifs related to plant growth and development. On the contrary, the number of HD-Zip 1 (the differentiation of the palisade mesophyll cells), AACA-motif (involved in endosperm-specific negative expression) and MSA-like (cell cycle regulation) elements were 8, 3 and 2 respectively. Additionally, RY-element (seed-specific regulation) and GCN4_motif (endosperm expression) were also identified in the promoters of the *CpbHLH* genes (**Figure 7**). The most common elements in phytohormone responsive category were ABRE (the abscisic acid-responsive element), CGTCA-motif and TGACG-motif (elements involved in MeJA responsiveness) and the TCA element (SA-responsive element) (**Figure 7**). In the last category, a lot of important CREs related to plant abiotic stress were detected. Most abundant of these were the ABRE (drought response element), ARE (anaerobic induced response element), MBS (drought induced response element) and LTR (low temperature response element). Other stress response CREs, such as GC-motif (anoxic specific inducibility element), TC-rich (defense and stress response element) and ERE elements (oxidative stress responsive elements were also identified (**Figure 7**).

### 3.6 Expression profiles of *CpbHLH* genes in salt stress under hydroponic experiment

**Figure 8:**
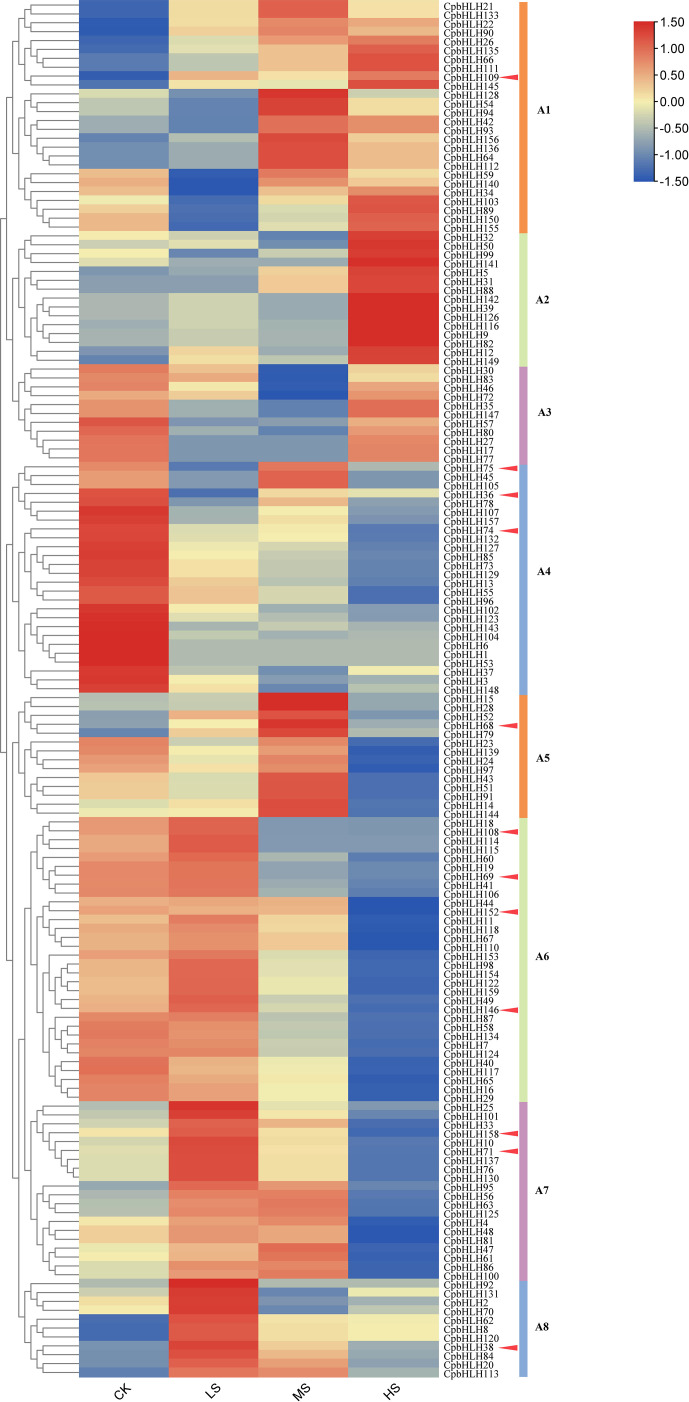
Clustering expression analysis of 159 *CpbHLH* genes in salt stress based on hydroponic experiments. The CK, LS, MS and HS represent the NaCl concentrations of 0%, 0.15%, 0.3% and 0.45% respectively. The transcript abundance level was normalized and hierarchically clustered by using the log 2 (FPKM + 1) comparison among genes of different treatments. The expression value is presented on the color scale, with red representing high expression and blue representing low expression. A1-A8 represent different clusters. In order to distinguish A1-A8 clusters more intuitively, lines of different colours were used in the right.

Analysis of gene expression profiles is an effective way to determine gene functions. Hence, the leaves of *C. paliurus* treated with different salt concentrations (0%, 0.15%, 0.3%, and 0.45% NaCl) for 30 days in hydroponic experiment were sequenced and analyzed (Zhang et al., 2021). The raw sequencing data were submitted to the NCBI BioProject database under project number PRJNA700136. The RPKM (Reads Per Kilobase per Million mapped reads) values of 159 *CpbHLH* genes were obtained from the transcriptome data to estimate the expression levels of bHLH family members. However, *CpbHLH119/121/138/151* were not analyzed because of the absence or low level of expression in the transcriptome data. **Figure 8** showed that 155 of these genes were expressed in all concentrations of NaCl treatments with different expression patterns, providing evidence that *CpbHLH* genes are significantly affected by salt stress.

Based on the similarity of expression patterns, the 155 *CpbHLH* genes were clustered into 8 clusters, named A1-A8 (**Figure 8**). The genes in cluster A1 were mainly expressed in the middle (0.30% NaCl) or high (0.45% NaCl) salinity condition and did not change significantly under low (0.15% NaCl) salinity condition. In contrast, *CpbHLHs* in cluster A6, A7 and A8 was strongly and preferentially expressed under low salt concentrations and down-regulated under high salt concentration. In cluster A2, the expression of *CpbHLH* genes did not change significantly under low and middle salt stress, but reached its highest value at high salinity treatment. However, expressions of most genes in cluster A4 varied with salt concentration treatments, and expression of these genes were all down-regulated under salt treatments and reached its lowest value at 0.45% NaCl treatment. However, very low expression levels of these genes in cluster A3 and A5 were observed at middle and high salt concentrations, respectively (**Figure 8**). In particular, among these 155 genes, the expression of some genes were strongly induced or inhibited under salt stress. For example, compared with the CK, the expressions of *CpbHLH36/74/75* in cluster A4 were down regulated by nearly folds of 3 in the low salinity treatment (0.15% NaCl), especially *CpbHLH74* down regulated by nearly folds of 9 in the high salinity treatment (0.45% NaCl). Similarly, seven differentially expressed genes (DEGs) (*CpbHLH68/69/71/108/146/152/158*) were identified in the A5, A6 and A7, indicating a response to salt stress (**Figure 8**).

### 3.7 Expression analysis of candidate genes in response to salt in pot experiment

**Figure 9:**
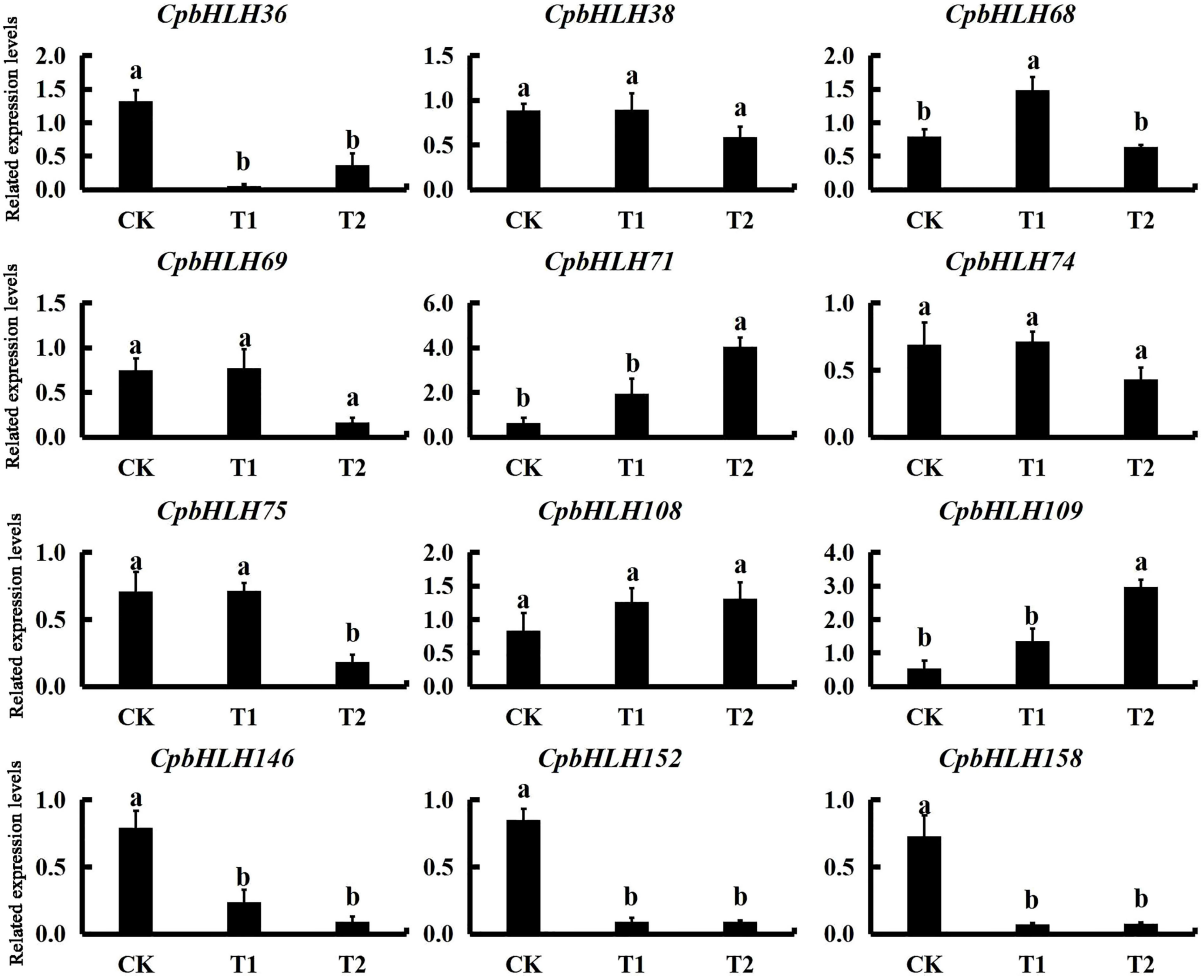
Expression profiles of the 12 candidate *CpbHLH* genes responding to salt stress treatments in pot experiment. The standard errors from three biological and three technical replications are presented as error bars. Following analysis of variance, significant differences identified by Duncan’s test (*p* < 0.05), using SPSS v.22, are represented by different letters.

Combining the results from both GO annotation and expression profiles analysis in hydroponic experiment, twelve salt-induced candidate genes (*CpbHLH36/38/68/69/71/74/75/108/109/146/152/158*) were selected for further qRT-PCR analysis using templates from pot experiment with three salt concentrations (0% NaCl, 0.2% NaCl and 0.4% NaCl) (**Figure 9**). Notably, eight candidate genes (*CpbHLH36/68/71/75/109/146/152/158*) were up regulated or decreased dramatically under different salt treatments, indicating that the expression of these genes was significantly induced or inhibited under salt stress (**Figure 9**). Among the eight genes, four genes (*CpbHLH36/146/152/158*) were down-regulated under salt stress, with three of these genes (*CpbHLH146/152/158*) being lowest expressed at 0.4% NaCl and one gene (*CpbHLH36*) being lowest expressed at 0.2% NaCl. On the contrary, three genes (*CpbHLH68/71/109*) were significantly induced by salt stress (**Figure 9**). In particular, three genes (*CpbHLH36/68/146*) responded strongly to salt treatments. Compared to the control, the variation trend of their expression in the pot experiment was highly consistent with that in the hydroponic experiment (**Figure 8**; **Figure 9**), indicating their vital functions in response to salt stress. For example, the expression level of *CpbHLH36* in both experiments was strongly inhibited under salt stress, whereas the inhibition degree was greater in low salt concentration than in high salt concentration.

### 3.8 Interaction network prediction of candidate genes

**Figure 10:**
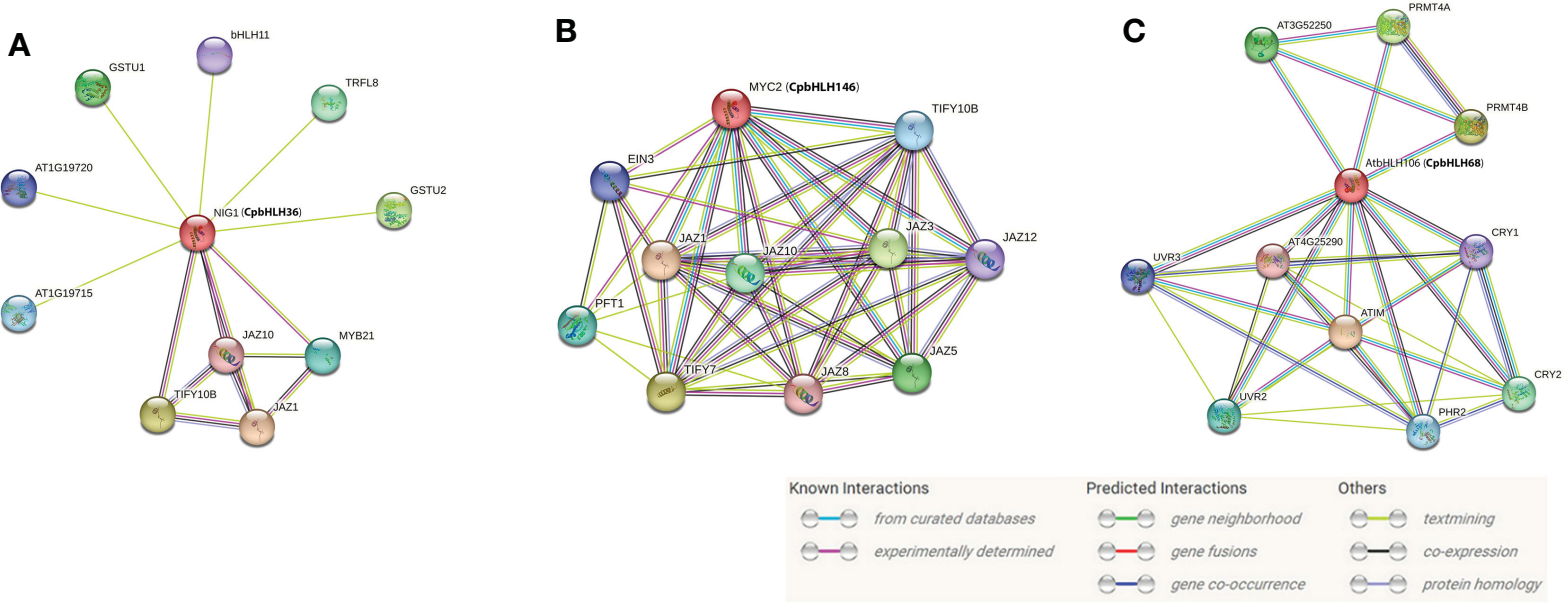
Interaction network analysis for *CpbHLH36* **(A)**, *CpbHLH146* **(B)** and *CpbHLH68* **(C)**. The predicted results are based on the orthologous gene in Arabidopsis. *CpbHLH* genes are shown in brackets.

It was reported that bHLH proteins exert regulatory effects by forming homodimers or heterodimers between bHLH proteins or between bHLH and non-bHLH proteins (Herold et al., 2002; Hernandez et al., 2007). Thus, the interaction network of three candidate genes was predicted by STRING (**Figure 10**), based on the *CpbHLH* homologous genes in *A. thaliana*. The investigation of *CpbHLH146* (*MYC2* ortholog) showed that it was involved in light, abscisic acid (ABA), and jasmonic acid (JA) signaling pathways and controlled additively subsets of JA-dependent responses with *MYC3* and *MYC4* (**Figure 10B**, **Supplementary Table S6**). Among the proteins interacting with *MYC2*, those related to JA signaling pathway accounted for the majority, including *JAZ1*, *JAZ3*, *JAZ5*, *JAZ8*, *JAZ10*, *JAZ12* and *TILY7*. Besides, *PFT1* was determined as phytochrome and flowering time regulatory protein and the *EIN3* probablely acted as a positive regulator in the ethylene response pathway (**Figure 10A**, **Supplementary Table S6**). The predicted network for *CpbHLH36* (*NIG* ortholog) showed that it plays central roles in regulating various proteins, and coincidently several of which were also involved in the jasmonic acid signaling pathway (*JAZ1* and *JAZ10*) (**Figure 10A**, **Supplementary Table S6**). Other proteins, *GSTU1* and *GSTU2*, could be involved in the conjugation of reduced glutathione to a wide number of exogenous and endogenous hydrophobic electrophiles and have a detoxification role against certain herbicides, whereas *bHLH11* and *TRFL8* both function in DNA binding (**Figure 10A**, **Supplementary Table S6**). Finally, the results of predicted network (**Figure 10C**, **Supplementary Table S6**) also indicated that *CpbHLH68* (ortholog of *bHLH106*) has crucial roles in DNA binding, whose function is the same as most of the proteins that interact with it. In addition, several interacting genes possibly regulate light responses, for example *CRY1* and *CPY2* are cryptochromes, and *UVR2* and *UVR3* involved in repair of UV radiation-induced DNA damage. However, *PRMT4B* has been identified as a positive regulator of oxidative stress tolerance that promotes the expression of antioxidant enzymes such as *APX1* and *GPX1* (**Figure 10A**, **Supplementary Table S6**). Overall, the results of the protein interaction network analysis indicated that the three candidate genes interact with proteins of various functions, making them crucial players in regulating plant growth and stress responses.

## 4 Discussion

### 4.1 Systematic and comprehensive genome-wide detection of *CpbHLHs* in *C paliurus*

Based on the whole genome of *C. paliurus*, 159 *bHLH* genes were systematically identified in the present study (**Supplementary Table S2**). The number of *CpbHLH* genes was the same as that identified in tomato (Sun et al., 2015), but smaller than that in Arabidopsis (162 genes) (Toledo-Ortiz et al., 2003) and apple (175 genes) (Yang et al., 2017), whereas greater than that in grape (94 genes) (Wang et al., 2018a), strawberry (113 genes) (Zhao et al., 2018) and jujube (92 genes) (Li et al., 2019). Overall, 159 CpbHLH proteins were further categorized into 26 subfamilies (**Figure 4**), according to the phylogenetic tree with the nomenclature protocol of bHLH proteins in *C. paliurus* and Arabidopsis (Heim et al., 2003), in agreement with results from previous studies (Pires and Dolan, 2010; Sun et al., 2015; Chu et al., 2018). However, the *CpbHLHs* were distributed almost evenly across 20 subfamilies, similar to *Camellia sinensis* (Sun et al., 2015) and *O. fragrans* (Li et al., 2020b). Moreover, our result indicated that no *CpbHLHs* were found in subfamily X, whereas the most *CpbHLH* members were detected in subfamily XII (**Figure 4**), with the number of members in this family increasing from 17 in Arabidopsis to 22 in *C. paliurus*. Differences in the numbers of *bHLH* genes among plant species may be due to gene replication events or genome size or gene loss during evolution (Flagel and Wendel, 2009; Li et al., 2020b).

Based on the analysis of the conserved motif and intron/exon (**Figure 5B, C**), the results showed that *CpbHLHs* in the same subfamily of the phylogenetic tree were similar in genetic and motif structures, further confirming the accuracy of subgroup classification of phylogenetic tree (**Figure 4**; **Figure 5**). Totally twenty motifs were identified in 159 CpbHLH proteins (**Figure 5B**). However, among them, motifs 1 and 2 existed in almost every CpbHLH protein and represented main components of the bHLH domain with high capability of conserved DNA binding, suggesting that the two motifs had very important implications about the functioning of *bHLH* genes (Zhang et al., 2020b). Nonetheless, the remaining 18 conserved non-bHLH domains can also feature separately in *CpbHLHs* in their respective subfamilies, similar to the other plant species (Chu et al., 2018; Li et al., 2020b). For example, most *bHLH* genes of subfamily III(d+e) in *Panax ginseng* (Chu et al., 2018) contained MYC-N structures (bHLH-MYC_N domain, Pfam : PF14215), which have been proved functioning in regulating the biosynthesis of phenylpropane. In this study, all *CpbHLHs* of III(d+e) also contained MYC-N structures (motif 5, 8, 10) (**Figure 5B**; **Supplementary Table S4**), implying that *CpbHLHs* of the same subgroup may have the similar roles. It was reported that gain/loss of exons and introns may result in the functional diversification of gene families (Xu et al., 2012a), whereas introns are related to gene evolution, and especieally the genes with few or no introns are more highly expressed in plants (Chung et al., 2006; Ren et al., 2006). In the present study, the intron-less *CpbHLHs* were distributed across subfamilies III (d+e) and VIIIb (**Figure 5C**), in accordance with the phenomenon in *P. ginseng* (Chu et al., 2018), apple (Yang et al., 2017) and Osmanthus (Li et al., 2020b), suggesting *CpbHLHs* of these subgroups could facilitates rapid and timely response to various stresses (Jeffares et al., 2008).

### 4.2 Functional prediction and identification of salt tolerance genes of *CpbHLHs*

Transcriptional regulation is a basic process of gene regulation in response to stress signals and a mass of TFs are involved in regulating plant responses to a given stress (Riechmann et al., 2000). The results of GO annotation in this study showed the functions of the *CpbHLH* genes are diverse (**Figure 6**; **Supplementary Table S5**), supporting that the bHLH TFs plays a crucial role in regulating plant growth, development and stress response (Shen et al., 2021). Several lines of evidence showed that salt stress had adverse effects on photosynthesis and the accumulation of secondary metabolites in *C. paliurus* (Zhang et al., 2021; Zhang et al., 2022a). Therefore, the detection of salt stress response genes from *CpbHLHs* will be helpful to achieve salt-tolerant breeding of *C. paliurus*.

The transcriptome sequencing analysis of salt treatments in the hydroponics provided specific expression data for the *CpbHLHs*, which makes it possible to further study the function of these genes. The RPKM values from our hydroponics showed that a large number of *CpbHLH* genes were induced/repressed under NaCl stress (**Figure 8**). According to the RPKM data, ten significantly differentially expressed genes (*CpbHLH36/68/69/71/74/75/108/146/152/158*) were predicted to function in responding to salt stress (**Figure 8**). Moreover, the molecular function annotations of 159 *CpbHLHs* indicated that three genes (*CpbHLH38/68/109*) strongly responded to salt stress (**Supplementary Table S5**). Thus, the 12 genes mentioned above were predicted to be candidate genes in response to salt stress and were selected for further qRT-PCR analysis, using salt-treated templates collected from our pot experiment. The qRT-PCR results showed that the expression of three genes (*CpbHLH36/68/146*) strongly responded to the salt treatments (**Figure 9**), and the variation trend of their expression levels was highly similar in the two salt stress experiments (**Figure 8**; **Figure 9**), indicating that these genes were specific for the regulation of salt tolerance in *C. paliurus*.

Phylogenetic analysis can be used to derive orthogonal relationships based on sequence similarity and protein structure, while the most closely related *bHLH* genes in the phylogenetic tree may share a similar function (Wang et al., 2021). The existed research indicated that *AtbHLH106* could enhance salt tolerance of plant by directly interacting with the G-box of salt tolerant genes (Ahmad et al., 2015), whereas the *CpbHLH68* was clustered in the same clade that possess high bootstrap value with *AtbHLH106* (**Figure 4**), suggesting *CpbHLH68* may be involved in response to salt stress. In addition, DNA sequences are decisive factors of the binding specificity between transcription factors and their genomic targets (Gordân et al., 2013), and our results from the DNA-binding ability of 159 *CpbHLHs* showed that *CpbHLH68* was G-box-binding protein (**Supplementary Table S3**), which further suggests that *CpbHLH68*, similar to *AtbHLH106*, may respond to salt stress by binding to G-box of target genes. Moreover, *AtbHLH6* (*ATMYC2*) has been reported to exhibit a significant response to salt and drought stresses (Abe et al., 1997; Aleman et al., 2016), while *AtNIG1* (a salt stress-responsive gene) was the first known TF participating in salt stress signal by binding calcium ions and bound to the E-box sequence (CANNTG) (Kim and Kim, 2006). Our study showed that *CpbHLH36* and *CpbHLH146* were clustered in the same clade with *AtbHLH6*(*MYC2*) and *AtbHLH28*(*AtNIG1*) (**Figure 4**), suggesting that *CpbHLH36* and *CpbHLH146* are also E-box proteins (**Supplementary Table S3**), and very likely to be involved in the regulation of salt stress signaling pathways.

In general, the function of a given gene can be inferred from its homologous genes (Yue et al., 2016; Qu et al., 2022). Therefore, Arabidopsis orthologs were used to predict the regulatory network of these three candidate genes (*CpbHLH36/68/146*) in this study. Some previous researches showed that *AtMYC2* was involved in the regulation of ABA-inducible genes under drought stress conditions (Gordân et al., 2013) and could provide a possible mechanistic link between ABA signaling and JA signaling (Abe et al., 1997; Zhang et al., 2021). The predicted interaction genes of *CpbHLH146* (*MYC2* ortholog) were mainly involved in the regulation of JA signaling (**Figure 10A**, **Supplementary Table S6**). The interaction of plant hormone ABA and JA played a major role in abiotic stress tolerance (Xiong et al., 2002; Zhang et al., 2012b) and ABA-dependent pathways the was one of important abiotic stress response signaling transduction pathways (Zhang et al., 2012a). The promoter region of most ABA regulatory genes contains many ABA responsive elements (Leonhardt et al., 2004; Yamaguchi-Shinozaki and Shinozaki, 2005; Fujita et al., 2011). In this study, a high occurrence of ABRE (ABA-responsive element) and CGTCA-motif (MeJA-responsive element) cis-acting elements was detected in the promoters of *CpbHLH146* (**Figure 7**). Thus, it can be inferred that this gene may have an important role in regulating stress resistance by regulating the expression of key genes in the ABA signaling pathway. Furthermore, most interaction genes of *CpbHLH36* (*AtNIG1* ortholog) and *CpbHLH68* (*bHLH106* ortholog) were mainly involved in DNA binding (**Figure 10A**, **Supplementary Table S6**), which further supports our hypothesis that these two genes regulate plant salt stress mainly *via* recognizing G-box of target genes. Moreover, *CpbHLH36* (*AtNIG1* ortholog) was also interacted with some JA signaling pathway proteins (**Figure 10A**, **Supplementary Table S6**), and it was in the same cluster of the phylogenetic tree with *CpbHLH146* (*MYC2* ortholog) (**Figure 4**). Besides, the similar expression trend of *CpbHLH36* was observed between pot experiment and hydroponic experiment, the same as to *CpbHLH146* (**Figure 8**; **Figure 9**). Therefore, it could be concluded that there is an indirect interaction between *CpbHLH36* and *CpbHLH146* at the protein level and these two genes coordinately control the expression of downstream genes, whereas the plant salt tolerance may depend upon the co-expression of these two genes.

In short, combined with the above results, *CpbHLH36/68/146* could be the key putative candidates in response to salt stress in *C. paliurus.* However, characterizations of these three genes involved in the regulation of salt tolerance varied. *CpbHLH36/68/146* are all G-box proteins, and may respond to salt stress by binding to G-box of target genes. Secondly, *CpbHLH36* may participate in salt stress signal by binding calcium ions and regulating the expression of key genes in the JA signaling pathway. Thirdly, *CpbHLH146* was very likely to be involved in the regulation of salt stress in ABA signaling pathways. Moreover, it is noted that there exists an indirect interaction between *CpbHLH36* and *CpbHLH146* at the protein level, thus we guess the salt tolerance of *C. paliurus* may depend upon the co-expression of these two genes.

In conclusion, it is the first report to identify the TF family based on the whole genome of *C. paliurus*. A total of 159 *CpbHLH* genes were detected and divided into 26 subfamilies, according to their evolutionary characteristics. In addition to investigating their structures and DNA-binding abilities, expression analysis from both the pot and hydroponic experiments and the regulatory network were also performed to determine which genes are most active for salt stress responses in this species. A total of 12 candidate genes were selected in response to salt stress, whereas the 3 genes (*CpbHLH36/68/146*) were further verified to be involved in regulating the salt tolerance of *C. paliurus* based on a pot experiment and protein interaction network analysis. Our findings would not only provide a basis for further understanding regulatory mechanisms of bHLH proteins TFs, but also drive progress in genetic improvement for the salt tolerance of *C. paliurus*.

## Data availability statement

The whole genome sequencing raw data including Illumina short reads, PacBio long reads, Hi-C interaction reads, and transcriptome data have been submitted to the Genome Sequence Archive at the National Genomics Data Center (NGDC), Beijing Institute of Genomics (BIG), Chinese Academy of Sciences (CAS) / China National Center for Bioinformation (CNCB) (GSA: CRA004671 and BioProject: PRJCA005987), and are publicly accessible at https://ngdc.cncb.ac.cn/gsa/.

## Author contributions

ZZ: Conceptualization, writing-original draft, visualization, data analysis, bioinformatics analysis. JF: Participated in the pot experiment. SF: Methodology, writing-review & editing, funding acquisition. LZ: Participated in the hydroponic experiment HJ: Participated in the pot experiment. All authors contributed to the article and approved the submitted version.

## References

[B1] Abe H. Yamaguchi-Shinozaki K. Urao T. Iwasaki T. Hosokawa D. Shinozaki K. (1997). Role of arabidopsis MYC and MYB homologs in drought- and abscisic acid-regulated gene expression. Plant Cell 9 (10), 1859–1868. doi: 10.1105/tpc.9.10.1859 9368419 PMC157027

[B2] Agarwal P. K. Agarwal P. Reddy M. K. Sopory S. K. (2006). Role of DREB transcription factors in abiotic and biotic stress tolerance in plants. Plant Cell Rep. 25 (12), 1263–1274. doi: 10.1007/s00299-006-0204-8 16858552

[B3] Ahmad A. Niwa Y. Goto S. Ogawa T. Shimizu M. Suzuki A.. (2015). bHLH106 integrates functions of multiple genes through their G-box to confer salt tolerance on arabidopsis. PloS One 10 (5), e0126872. doi: 10.1371/journal.pone.0126872 25978450 PMC4433118

[B4] Aleman F. Yazaki J. Lee M. Takahashi Y. Kim A. Y. Li Z.. (2016). An ABA-increased interaction of the PYL6 ABA receptor with MYC2 transcription factor: A putative link of ABA and JA signaling. Sci. Rep. 6, 28941. doi: 10.1038/srep28941 27357749 PMC4928087

[B5] Atchley W. R. Terhalle W. Dress A. (1999). Positional dependence, cliques, and predictive motifs in the bHLH protein domain. J. Mol. Evol. 48 (5), 501–516. doi: 10.1007/PL00006494 10198117

[B6] Babitha K. C. Ramu S. V. Pruthvi V. Mahesh P. Nataraja K. N. Udayakumar M. (2013). Co-Expression of *AtbHLH17* and *AtWRKY28* confers resistance to abiotic stress in arabidopsis. Transgenic Res. 22 (2), 327–341. doi: 10.1007/s11248-012-9645-8 22948308

[B7] Bailey T. L. Boden M. Buske F. A. Frith M. Grant C. E. Clementi L.. (2009). MEME SUITE: tools for motif discovery and searching. Nucleic Acids Res. 37, W202–W208. doi: 10.1093/nar/gkp335 19458158 PMC2703892

[B8] Bhatnagar-Mathur P. Vadez V. Sharma K. K. (2008). Transgenic approaches for abiotic stress tolerance in plants: retrospect and prospects. Plant Cell Rep. 27 (3), 411–424. doi: 10.1007/s00299-007-0474-9 18026957

[B9] Carretero-Paulet L. Galstyan A. Roig-Villanova I. Martínez-García J. F. Bilbao-Castro J. R. Robertson D. L. (2010). Genome-wide classification and evolutionary analysis of the bHLH family of transcription factors in arabidopsis, poplar, rice, moss, and algae. Plant Physiol. 153 (3), 1398–1412. doi: 10.1104/pp.110.153593 20472752 PMC2899937

[B10] Chen C. Chen H. Zhang Y. Thomas H. R. Frank M. H. He Y.. (2020). TBtools: an integrative toolkit developed for interactive analyses of big biological data. Mol. Plant 13 (8), 1194–1202. doi: 10.1016/j.molp.2020.06.009 32585190

[B11] Chen X. Mao X. Huang P. Fang S. (2019). Morphological characterization of flower buds development and related gene expression profiling at bud break stage in heterodichogamous *Cyclocarya paliurus* (Batal.) lljinskaja. Genes (Basel) 10(10):818. doi: 10.3390/genes10100818 PMC6827045 31627470

[B12] Chen C. Xia R. Chen H. He Y. (2018). TBtools, a toolkit for biologists integrating various HTS-data handling tools with a user-friendly interface. bioRxiv. doi: 10.1101/289660

[B13] Chen P. Yang W. Minxue W. Jin S. Liu Y. (2021). Hydrogen sulfide alleviates salinity stress in *Cyclocarya paliurus* by maintaining chlorophyll fluorescence and regulating nitric oxide level and antioxidant capacity. Plant Physiol. Biochem. 167, 738–747. doi: 10.1016/j.plaphy.2021.09.004 34509132

[B14] Chinnusamy V. Ohta M. Kanrar S. Lee B. H. Hong X. Agarwal M.. (2003). ICE1: a regulator of cold-induced transcriptome and freezing tolerance in arabidopsis. Genes Dev. 17 (8), 1043–1054. doi: 10.1101/gad.1077503 12672693 PMC196034

[B15] Chu Y. Xiao S. Su H. Liao B. Zhang J. Xu J.. (2018). Genome-wide characterization and analysis of bHLH transcription factors in *Panax ginseng* . Acta Pharm. Sin. B. 8 (4), 666–677. doi: 10.1016/j.apsb.2018.04.004 30109190 PMC6089850

[B16] Chung B. Simons C. Firth A. Brown C. Hellens R. (2006). Effect of 5’UTR introns on gene expression in *Arabidopsis thaliana* . BMC Genomics 7, 120. doi: 10.1186/1471-2164-7-120 16712733 PMC1482700

[B17] Conesa A. Götz S. García-Gómez J. M. Terol J. Talón M. Robles M. (2005). Blast2GO: a universal tool for annotation, visualization and analysis in functional genomics research. Bioinformatics 21 (18), 3674–3676. doi: 10.1093/bioinformatics/bti610 16081474

[B18] Cui J. You C. Zhu E. Huang Q. Ma H. Chang F. (2016). Feedback regulation of DYT1 by interactions with downstream bHLH factors promotes DYT1 nuclear localization and anther development. Plant Cell 28 (5), 1078–1093. doi: 10.1105/tpc.15.00986 27113773 PMC4904671

[B19] Fang S. (2022). A review on the development history and the resource silviculture of *Cyclocarya paliurus* industry. J. Nanjing For. Univ. (Nat. Sci. Ed.) 46 (6), 115–126. doi: 10.12302/j.issn.1000-2006.202206019

[B20] Fang S. Wang J. Wei Z. Zhu Z. (2006). Methods to break seed dormancy in *Cyclocarya paliurus* (Batal)Iljinskaja. Sci. Hortic. 110 (3), 305–309. doi: 10.1016/j.scienta.2006.06.031

[B21] Fang S. Yang W. Chu X. Shang X. She C. Fu X. (2011). Provenance and temporal variations in selected flavonoids in leaves of *Cyclocarya paliurus* . Food Chem. 124 (4), 1382–1386. doi: 10.1016/j.foodchem.2010.07.095

[B22] Feller A. Machemer K. Braun E. L. Grotewold E. (2011). Evolutionary and comparative analysis of MYB and bHLH plant transcription factors. Plant J. 66 (1), 94–116. doi: 10.1111/j.1365-313X.2010.04459.x 21443626

[B23] Feng H. L. Ma N. N. Meng X. Zhang S. Wang J. R. Chai S.. (2013). A novel tomato MYC-type ICE1-like transcription factor, SlICE1a, confers cold, osmotic and salt tolerance in transgenic tobacco. Plant Physiol. Biochem. 73, 309–320. doi: 10.1016/j.plaphy.2013.09.014 24184451

[B24] Finn R. D. Bateman A. Clements J. Coggill P. Eberhardt R. Y. Eddy S. R. (2014). Pfam: the protein families database. Nucleic Acids Res. 42, 222 230. doi: 10.1093/nar/gkt1223 PMC3965110 24288371

[B25] Flagel L. E. Wendel J. F. (2009). Gene duplication and evolutionary novelty in plants. New Phytol. 183 (3), 557–564. doi: 10.1111/j.1469-8137.2009.02923.x 19555435

[B26] Fujita Y. Fujita M. Shinozaki K. Yamaguchi-Shinozaki K. (2011). ABA-mediated transcriptional regulation in response to osmotic stress in plants. J. Plant Res. 124 (4), 509–525. doi: 10.1007/s10265-011-0412-3 21416314

[B27] Gordân R. Shen N. Dror I. Zhou T. Horton J. Rohs R.. (2013). Genomic regions flanking e-box binding sites influence DNA binding specificity of bHLH transcription factors through DNA shape. Cell Rep. 3 (4), 1093–1104. doi: 10.1016/j.celrep.2013.03.014 23562153 PMC3640701

[B28] Heim M. A. Jakoby M. Werber M. Martin C. Weisshaar B. Bailey P. C. (2003). The basic helix-loop-helix transcription factor family in plants: a genome-wide study of protein structure and functional diversity. Mol. Biol. Evol. 20 (5), 735–747. doi: 10.1093/molbev/msg088 12679534

[B29] Hernandez J. M. Feller A. Morohashi K. Frame K. Grotewold E. (2007). The basic helix loop helix domain of maize r links transcriptional regulation and histone modifications by recruitment of an EMSY-related factor. Proc. Natl. Acad. Sci. U.S.A. 104 (43), 17222–17227. doi: 10.1073/pnas.0705629104 17940002 PMC2040437

[B30] Herold S. Wanzel M. Beuger V. Frohme C. Beul D. Hillukkala T.. (2002). Negative regulation of the mammalian UV response by myc through association with miz-1. Mol. Cell 10 (3), 509–521. doi: 10.1016/S1097-2765(02)00633-0 12408820

[B31] Higo K. Ugawa Y. Iwamoto M. Korenaga T. (1999). Plant cis-acting regulatory DNA elements (PLACE) database: 1999. Nucleic Acids Res. 27 (1), 297–300. doi: 10.1093/nar/27.1.297 9847208 PMC148163

[B32] Hou X. J. Li J. M. Liu B. L. Wei L. (2017). Co-Expression of basic helix–loop–helix protein (bHLH) and transcriptional activator-myb genes induced anthocyanin biosynthesis in hairy root culture of *Nicotiana tabacum l* and *Ipomea tricolor* . Acta Physiol. Plant 39 (2), 59. doi: 10.1007/s11738-017-2362-4

[B33] Jeffares D. C. Penkett C. J. Bähler J. (2008). Rapidly regulated genes are intron poor. Trends Genet. 24 (8), 375–378. doi: 10.1016/j.tig.2008.05.006 18586348

[B34] Ji X. Nie X. Liu Y. Zheng L. Zhao H. Zhang B.. (2016). A bHLH gene from *Tamarix hispida* improves abiotic stress tolerance by enhancing osmotic potential and decreasing reactive oxygen species accumulation. Tree Physiol. 36 (2), 193–207. doi: 10.1093/treephys/tpv139 26786541

[B35] Jiang Y. Yang B. Deyholos M. K. (2009). Functional characterization of the arabidopsis bHLH92 transcription factor in abiotic stress. Mol. Genet. Genom. 282 (5), 503–516. doi: 10.1007/s00438-009-0481-3 19760256

[B36] Johnson L.S. Eddy S.R. Portugaly E. (2010). Hidden Markov model speed heuristic anditerative HMM search procedure. BMC Bioinformatics 11, 431. doi: 10.1186/1471-2105-11-431 20718988 PMC2931519

[B37] Katiyar A. Smita S. Lenka S. K. Rajwanshi R. Chinnusamy V. Bansal K. C. (2012). Genome-wide classification and expression analysis of MYB transcription factor families in rice and arabidopsis. BMC Genom. 13, 544. doi: 10.1186/1471-2164-13-544 PMC3542171 23050870

[B38] Kavas M. Baloğlu M. C. Atabay E. S. Ziplar U. T. Daşgan H. Y. Ünver T. (2016). Genome-wide characterization and expression analysis of common bean bHLH transcription factors in response to excess salt concentration. Mol. Genet. Genom. 291 (1), 129–143. doi: 10.1007/s00438-015-1095-6 26193947

[B39] Kim J. Kim H. Y. (2006). Functional analysis of a calcium-binding transcription factor involved in plant salt stress signaling. FEBS Lett. 580 (22), 5251–5256. doi: 10.1016/j.febslet.2006.08.050 16962584

[B40] Kumar S. Stecher G. Li M. Knyaz C. Tamura K. (2018). MEGA X: Molecular evolutionary genetics analysis across computing platforms. Mol. Biol. Evol. 35 (6), 1547–1549. doi: 10.1093/molbev/msy096 29722887 PMC5967553

[B41] Kurihara H. Fukami H. Kusumoto A. Toyoda Y. Shibata H. Matsui Y.. (2003). Hypoglycemic action of *Cyclocarya paliurus* (Batal.) iljinskaja in normal and diabetic mice. Biosci. Biotechnol. Biochem. 67 (4), 877–880. doi: 10.1271/bbb.67.877 12784631

[B42] Leonhardt N. Kwak J. M. Robert N. Waner D. Leonhardt G. Schroeder J. I. (2004). Microarray expression analyses of arabidopsis guard cells and isolation of a recessive abscisic acid hypersensitive protein phosphatase 2C mutant[W]. Plant Cell 16 (3), 596–615. doi: 10.1105/tpc.019000 14973164 PMC385275

[B43] Letunic I. Khedkar S. Bork P. (2021). SMART: recent updates, new developments and status in 2020. Nucleic Acids Res. 49 (D1), D458–d460. doi: 10.1093/nar/gkaa937 33104802 PMC7778883

[B44] Li H. Gao W. Xue C. Zhang Y. Liu Z. Zhang Y.. (2019). Genome-wide analysis of the bHLH gene family in Chinese jujube (*Ziziphus jujuba* mill.) and wild jujube. BMC Genom. 20 (1), 568. doi: 10.1186/s12864-019-5936-2 PMC6617894 31291886

[B45] Li Y. Li L. Ding W. Li H. Shi T. Yang X.. (2020b). Genome-wide identification of *Osmanthus fragrans* bHLH transcription factors and their expression analysis in response to abiotic stress. Environ. Exp. Bot. 172. doi: 10.1016/j.envexpbot.2020.103990

[B46] Li H. Shi J. Wang Z. Zhang W. Yang H. (2020a). H_2_S pretreatment mitigates the alkaline salt stress on malus hupehensis roots by regulating Na(^+^)/K(^+^) homeostasis and oxidative stress. Plant Physiol. Biochem. 156, 233–241. doi: 10.1016/j.plaphy.2020.09.009 32977178

[B47] Li M. Sun L. Gu H. Cheng D. Guo X. Chen R.. (2021). Genome-wide characterization and analysis of bHLH transcription factors related to anthocyanin biosynthesis in spine grapes (*Vitis davidii*). Sci. Rep. 11 (1), 6863. doi: 10.1038/s41598-021-85754-w 33767241 PMC7994560

[B48] Li H. Sun J. Xu Y. Jiang H. Wu X. Li C. (2007). The bHLH-type transcription factor AtAIB positively regulates ABA response in arabidopsis. Plant Mol. Biol. 65 (5), 655–665. doi: 10.1007/s11103-007-9230-3 17828375

[B49] Liao Y. Zhang P. Zhang Q. Li X. (2022). Advances in salt-tolerant mechanisms of trees and forestation techniques on saline-alkali land. J. Nanjing For. Univ. (Nat. Sci. Ed.) 46 (6), 96–104. doi: 10.12302/j.issn.1000-2006.202209010

[B50] Lim S. H. Kim D. H. Kim J. K. Lee J. Y. Ha S. H. (2017). A radish basic helix-Loop-Helix transcription factor, RsTT8 acts a positive regulator for anthocyanin biosynthesis. Front. Plant Sci. 8. doi: 10.3389/fpls.2017.01917 PMC5682339 29167678

[B51] Manchester S. R. Chen Z. D. Lu A. M. Uemura K. (2009). Eastern Asian Endemic seed plant genera and their paleogeographic history throughout the northern hemisphere. J. Syst. Evol. 47, 1–2. doi: 10.1111/j.1759-6831.2009.00001.x

[B52] Mantri N. Patade V. Penna S. Ford R. Pang E. (2012a). Abiotic stress responses in plants: Present and future. Springer New York 1-19. doi: 10.1007/978-1-4614-0634-1_1

[B53] Mao K. Dong Q. Li C. Liu C. Ma F. (2017). Genome wide identification and characterization of apple bHLH transcription factors and expression analysis in response to drought and salt stress. Front. Plant Sci. 8. doi: 10.3389/fpls.2017.00480 PMC5387082 28443104

[B54] Marchler-Bauer A. Bryant S. H. (2004). CD-Search: protein domain annotations on the fly. Nucleic Acids Res. 32, W327–W331. doi: 10.1093/nar/gkh454 15215404 PMC441592

[B55] Penfield S. (2001). MYB61 is required for mucilage deposition and extrusion in the arabidopsis seed coat. Plant Cell 13, 2777–2791. doi: 10.1105/tpc.13.12.2777 11752387 PMC139488

[B56] Pires N. Dolan L. (2010). Origin and diversification of basic-helix-loop-helix proteins in plants. Mol. Biol. Evol. 27 (4), 862–874. doi: 10.1093/molbev/msp288 19942615 PMC2839125

[B57] Qin J. Yue X. Fang S. Qian M. Zhou S. Shang X.. (2021). Responses of nitrogen metabolism, photosyntheticparameter and growth to nitrogen fertilization in *Cyclocarya paliurus* . For. Ecol. Manage. 502, 119715. doi: 10.1016/j.foreco.2021.119715

[B58] Qu Y. Chen X. Mao X. Huang P. Fu X. (2022). Transcriptome analysis reveals the role of GA_3_ in regulating the asynchronism of floral bud differentiation and development in heterodichogamous *Cyclocarya paliurus* (Batal.) iljinskaja. Int. J. Mol. Sci. 23, 6763. doi: 10.3390/ijms23126763 35743203 PMC9224186

[B59] Ren X.-Y. Vorst O. Fiers M. W. E. J. Stiekema W. J. Nap J.-P. (2006). In plants, highly expressed genes are the least compact. Trends Genet. 22 (10), 528–532. doi: 10.1016/j.tig.2006.08.008 16934358

[B60] Riechmann J. L. Heard J. Martin G. Reuber L. Jiang C. Keddie J.. (2000). Arabidopsis transcription factors: genome-wide comparative analysis among eukaryotes. Science 290 (5499), 2105–2110. doi: 10.1126/science.290.5499.2105 11118137

[B61] Shen T. Wen X. Wen Z. Qiu Z. Hou Q. Li Z.. (2021). Genome-wide identification and expression analysis of bHLH transcription factor family in response to cold stress in sweet cherry (*Prunus avium* l.). Sci. Hortic. 279, 109905. doi: 10.1016/j.scienta.2021.109905

[B62] Sorensen A. M. Kröber S. Unte U. S. Huijser P. Dekker K. Saedler H. (2003). The *Arabidopsis ABORTED MICROSPORES (AMS)* gene encodes a MYC class transcription factor. Plant J. 33 (2), 413–423. doi: 10.1046/j.1365-313x.2003.01644.x 12535353

[B63] Sum J. Guo Y. Li S. Zhou C. Chiang V. Li W. (2021). A functional study of bHLH106 transcription factor based on CRISPR/Cas9 in *Populus trichocarpa* . J. Nanjing For. Univ. (Nat. Sci. Ed.) 45 (6), 15–23. doi: 10.12302/j.issn.1000-2006.202107031

[B64] Sun H. Fan H. J. Ling H. Q. (2015). Genome-wide identification and characterization of the bHLH gene family in tomato. BMC Genom. 16 (1), 9. doi: 10.1186/s12864-014-1209-2 PMC4312455 25612924

[B65] Szklarczyk D. Gable A. L. Lyon D. Junge A. Wyder S. Huerta-Cepas J.. (2019). STRING v11: protein-protein association networks with increased coverage, supporting functional discovery in genome-wide experimental datasets. Nucleic Acids Res. 47 (D1), D607–d613. doi: 10.1093/nar/gky1131 30476243 PMC6323986

[B66] Thompson J. D. Gibson T. J. Plewniak F. Jeanmougin F. Higgins D. G. (1997). The CLUSTAL_X windows interface: flexible strategies for multiple sequence alignment aided by quality analysis tools. Nucleic Acids Res. 25 (24), 4876–4882. doi: 10.1093/nar/25.24.4876 9396791 PMC147148

[B67] Toledo-Ortiz G. Huq E. Quail P. H. (2003). The arabidopsis basic/helix-loop-helix transcription factor family. Plant Cell 15 (8), 1749–1770. doi: 10.1105/tpc.013839 12897250 PMC167167

[B68] Wang P. Su L. Gao H. Jiang X. Wu X. Li Y.. (2018a). Genome-wide characterization of bHLH genes in grape and analysis of their potential relevance to abiotic stress tolerance and secondary metabolite biosynthesis. Front. Plant Sci. 9. doi: 10.3389/fpls.2018.00064 PMC5799661 29449854

[B69] Wang Y. Zhang Y. Fan C. Wei Y. Meng J. Li Z.. (2021). Genome-wide analysis of MYB transcription factors and their responses to salt stress in *Casuarina equisetifolia* . BMC Plant Biol. 21 (1), 328. doi: 10.1186/s12870-021-03083-6 34238224 PMC8265015

[B70] Wang R. Zhao P. Kong N. Lu R. Pei Y. Huang C.. (2018b). Genome-wide identification and characterization of the potato bHLH transcription factor family. Genes (Basel) 9(1):54. doi: 10.3390/genes9010054 PMC5793205 29361801

[B71] Wu J. Y. Wilf P. Ding S. T. An P. C. Dai J. (2017). Late miocene *Cyclocarya* (Juglandaceae) from southwest China and its biogeographic implications. Int. J. Plant Sci. 178, 580–591. doi: 10.1086/692765

[B72] Xiong L. Schumaker K. S. Zhu J. K. (2002). Cell signaling during cold, drought, and salt stress. Plant Cell 14 Suppl (Suppl), S165–S183. doi: 10.1105/tpc.000596 12045276 PMC151254

[B73] Xu G. Guo C. Shan H. Kong H. (2012b). Divergence of duplicate genes in exon-intron structure. Proc. Natl. Acad. Sci. U.S.A. 109 (4), 1187–1192. doi: 10.1073/pnas.1109047109 22232673 PMC3268293

[B74] Xu B. Sathitsuksanoh N. Tang Y. Udvardi M. Zhang J.-Y. Shen Z.. (2012a). Overexpression of AtLOV1 in switchgrass alters plant architecture, lignin content, and flowering time. PloS One 7, e47399. doi: 10.1371/journal.pone.0047399 23300513 PMC3530547

[B75] Yamaguchi-Shinozaki K. Shinozaki K. (2005). Organization of cis-acting regulatory elements in osmotic- and cold-stress-responsive promoters. Trends Plant Sci. 10 (2), 88–94. doi: 10.1016/j.tplants.2004.12.012 15708346

[B76] Yang J. Gao M. Huang L. Wang Y. van Nocker S. Wan R.. (2017). Identification and expression analysis of the apple (*Malus × domestica*) basic helix-loop-helix transcription factor family. Sci. Rep. 7 (1), 28. doi: 10.1038/s41598-017-00040-y 28174429 PMC5428380

[B77] Yao X. Lin Z. Jiang C. Gao M. Wang Q. Yao N.. (2015). *Cyclocarya paliurus* prevents high fat diet induced hyperlipidemia and obesity in sprague-dawley rats. Can. J. Physiol. Pharmacol. 93 (8), 677–686. doi: 10.1139/cjpp-2014-0477 26203820

[B78] Yue H. Wang M. Liu S. Du X. Song W. Nie X. (2016). Transcriptome-wide identification and expression profiles of the WRKY transcription factor family in broomcorn millet (*Panicum miliaceum* l.). BMC Genomics 17, 343. doi: 10.1186/s12864-016-2677-3 27165545 PMC4862231

[B79] Zhai L. X. Ning Z. W. Huang T. Wen B. Liao C. H. Lin C. Y.. (2018). *Cyclocarya paliurus* leaves tea improves dyslipidemia in diabetic mice: a lipidomics-based network pharmacology study. Front. Pharmacol. 9, 973. doi: 10.3389/fphar.2018.00973 30210345 PMC6121037

[B80] Zhai Y. Zhang L. Xia C. Fu S. Zhao G. Jia J.. (2016). The wheat transcription factor, TabHLH39, improves tolerance to multiple abiotic stressors in transgenic plants. Biochem. Biophys. Res. Commun. 473 (4), 1321–1327. doi: 10.1016/j.bbrc.2016.04.071 27091431

[B81] Zhang T. Bai Y. Qi X. Yu X. Fang H. Li L.. (2022a). Cloning and expression analyses of *MhWRKY57* response to jasmonic acid in *Mentha canadensis* . J. Nanjing For. Univ. (Nat. Sci. Ed.) 46 (6), 279–287. doi: 10.12302/j.issn.1000-2006.202109036

[B82] Zhang Y. Gao W. Li H. Wang Y. Li D. Xue C.. (2020b). Genome-wide analysis of the bZIP gene family in Chinese jujube (*Ziziphus jujuba* mill.). BMC Genomics 21 (1), 483. doi: 10.1186/s12864-020-06890-7 32664853 PMC7362662

[B83] Zhang M. Liu Y. Han G. Zhang Y. Wang B. Chen M. (2020a). Salt tolerance mechanisms in trees: research progress. Trees 35 (3), 717–730. doi: 10.1007/s00468-020-02060-0

[B84] Zhang Z. Liu X. Wang X. Zhou M. Zhou X. Ye X.. (2012b). An R2R3 MYB transcription factor in wheat, TaPIMP1, mediates host resistance to *Bipolaris sorokiniana* and drought stresses through regulation of defense- and stress-related genes. New Phytol. 196 (4), 1155–1170. doi: 10.1111/j.1469-8137.2012.04353.x 23046089

[B85] Zhang L. Zhang Z. Fang S. Liu Y. Shang X. (2021). Integrative analysis of metabolome and transcriptome reveals molecular regulatory mechanism of flavonoid biosynthesis in *Cyclocarya paliurus* under salt stress. Ind. Crops Prod. 170. doi: 10.1016/j.indcrop.2021.113823

[B86] Zhang L. Zhang Z. Fang S. Liu Y. Shang X. (2022a). Metabolome and transcriptome analyses unravel the molecular regulatory mechanisms involved in photosynthesis of *Cyclocarya paliurus* under salt stress. Int. J. Mol. Sci. 23(3):1161. doi: 10.3390/ijms23031161 PMC8835658 35163101

[B87] Zhang Z. Zhang L. Liu Y. Shang X. Fang S. (2022b). Identification and expression analysis of R2R3-MYB family genes associated with salt tolerance in *Cyclocarya paliurus* . Int. J. Mol. Sci. 23(7):3429. doi: 10.3390/ijms23073429 PMC8998414 35408785

[B88] Zhang L. Zhao G. Jia J. Liu X. Kong X. (2012a). Molecular characterization of 60 isolated wheat *MYB* genes and analysis of their expression during abiotic stress. J. Exp. Bot. 63 (1), 203–214. doi: 10.1093/jxb/err264 21934119 PMC3245462

[B89] Zhao F. Li G. Hu P. Zhao X. Li L. Wei W.. (2018). Identification of basic/helix-loop-helix transcription factors reveals candidate genes involved in anthocyanin biosynthesis from the strawberry white-flesh mutant. Sci. Rep. 8(1):2721. doi: 10.1038/s41598-018-21136-z 29426907 PMC5807450

[B90] Zhao Q. Ren Y. R. Wang Q. J. Yao Y. X. You C. X. Hao Y. J. (2016). Overexpression of *MdbHLH104* gene enhances the tolerance to iron deficiency in apple. Plant Biotechnol. J. 14 (7), 1633–1645. doi: 10.1111/pbi.12526 26801352 PMC5066684

[B91] Zhou M. M. Quek S. Y. Shang X. L. Fang S. Z. (2021). Geographical variations of triterpenoid contents in *Cyclocarya paliurus* leaves and their inhibitory effects on hela cells. Ind. Crop Prod. 162, 113314. doi: 10.1016/j.indcrop.2021.113314

