# Supplementary material for: Genome-wide identification of bHLH transcription factors and their response to salt stress in Cyclocarya paliurus
Source: Front Plant Sci. 2023 Mar 9;14:1117246. doi: 10.3389/fpls.2023.1117246 (PMC10035414; doi:10.3389/fpls.2023.1117246)
Supplement: Supplementary file 1 [file DataSheet_1.zip › Supplementary Tables S1-S4.docx]

Supplementary Materials

| **Gene ID** | **Primer sequences** | **Length**/bp |
| --- | --- | --- |
| CpbHLH38-FP | 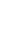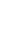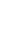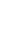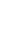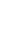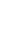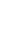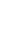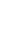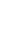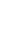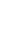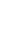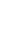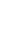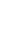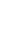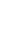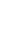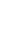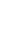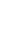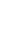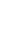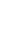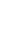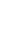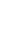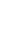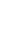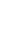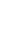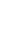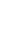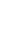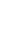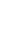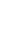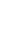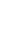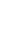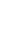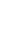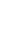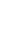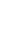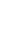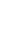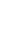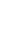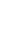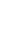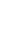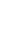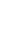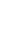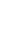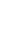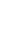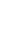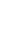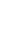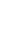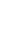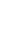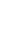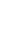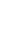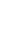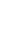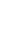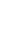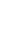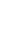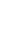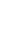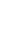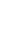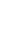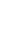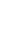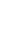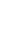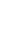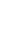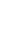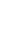GGTTGGCTTATTGCTGGGCT | 20 |
| CpbHLH38-FR | TAGACTCCTCCTCCACGACA | 20 |
| CpbHLH75-FP | 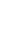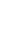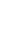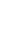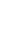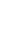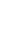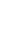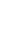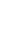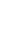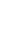AGGTGGGTGTGGTTGAGTTG | 20 |
| CpbHLH75-FR | GCCGCTGGTAAAGGATGTGT | 20 |
| CpbHLH74-FP | TCGCTAACCCGCTCTTTCTC | 20 |
| CpbHLH74-FR | AAGGACTTTGCTTGCTGGAC | 20 |
| CpbHLH146-FP | GGGTAGTCGGTTTTACACGCA | 21 |
| CpbHLH146-FR | CCCCATTTTCCCAGTTCCCA | 20 |
| CpbHLH36-FP | TACCTTCGCCTTCGCTGTC | 19 |
| CpbHLH36-FR | CCCCACCAATGCTCTGTTCT | 20 |
| CpbHLH152-FP | CCTCACCTACCTCCACCCTC | 20 |
| CpbHLH152-FR | GGTTTTTGGTTCGGTTCGCC | 20 |
| CpbHLH109-FP | ATTCGCCACCGCAGGATAGA | 20 |
| CpbHLH109-FR | GTCTTCACAGCACAACGAGG | 20 |
| CpbHLH158-FP | GCTCTCCCATCGTCGTCTTC | 20 |
| CpbHLH158-FR | TGGACTGCTCCTGCTCTTTG | 20 |
| CpbHLH71-FP | CCATCTCGCTTCGTCTGCT | 19 |
| CpbHLH71-FR | CGTTTGCTTGGTGGGGTTG | 19 |
| CpbHLH69-FP | TACTCCTCTCTCCGTTCGCT | 20 |
| CpbHLH69-FR | ATCAGTCCCACCACTTGCTT | 20 |
| CpbHLH68-FP | AGCCTGCCGAAAACCAAGA | 19 |
| CpbHLH68-FR | CTATCCTGCGGTGGCGTATC | 20 |
| CpbHLH108-FP | GGATTTCAAGGCAGGTAGGTCA | 22 |
| CpbHLH108-FR | CTGGAGTCTTCTGGTCTTCACA | 22 |
| 18s-FP | GTATGGTCGCAAGGCTGAAA | 20 |
| 18s-FR | CAGACAAATCGCTCCACCAA | 20 |

**Supplementary Table S1. Information of primer sequences**

**Supplementary Table S2.** Physical and chemical property prediction and location of bHLH family in *C. paliurus*

| **Gene ID** | **Molecular weight (Mw:Da)** | **Isoelectric point (pI)** |
| --- | --- | --- |
| CpbHLH1 | 36964.01 | 5.67 |
| CpbHLH2 | 29862.98 | 6.14 |
| CpbHLH3 | 10589.88 | 7.94 |
| CpbHLH4 | 37197.14 | 5.96 |
| CpbHLH5 | 59486.92 | 5.64 |
| CpbHLH6 | 50004.17 | 6.19 |
| CpbHLH7 | 48565.55 | 5.68 |
| CpbHLH8 | 37930.52 | 7.06 |
| CpbHLH9 | 43792.02 | 5.81 |
| CpbHLH10 | 56080.98 | 6.02 |
| CpbHLH11 | 31323.44 | 5.9 |
| CpbHLH12 | 60185 | 8.72 |
| CpbHLH13 | 23945.12 | 6.1 |
| CpbHLH14 | 42445.94 | 6.89 |
| CpbHLH15 | 26662.33 | 6.76 |
| CpbHLH16 | 60125.41 | 5.81 |
| CpbHLH17 | 26199.61 | 6.54 |
| CpbHLH18 | 27088.87 | 6.61 |
| CpbHLH19 | 27705.45 | 8.86 |
| CpbHLH20 | 64633.1 | 4.96 |
| CpbHLH21 | 37926.08 | 6.19 |
| CpbHLH22 | 30067.52 | 6.01 |
| CpbHLH23 | 59075.11 | 5.27 |
| CpbHLH24 | 38701.57 | 5.96 |
| CpbHLH25 | 38666.19 | 5.91 |
| CpbHLH26 | 41075.39 | 5.61 |
| CpbHLH27 | 28680.91 | 9.52 |
| CpbHLH28 | 62228.53 | 8 |
| CpbHLH29 | 31192.17 | 6.11 |
| CpbHLH30 | 24419.02 | 4.97 |
| CpbHLH31 | 49086.42 | 6.07 |
| CpbHLH32 | 25828.32 | 6.63 |
| CpbHLH33 | 70997.1 | 5.33 |
| CpbHLH34 | 54466.52 | 6.91 |
| CpbHLH35 | 34457.54 | 9.47 |
| CpbHLH36 | 74115.41 | 5.44 |
| CpbHLH37 | 62045.09 | 8.63 |
| CpbHLH38 | 27529.07 | 5.67 |
| CpbHLH39 | 10454.72 | 7.96 |
| CpbHLH40 | 32341.85 | 5.96 |
| CpbHLH41 | 39783.06 | 8.11 |
| CpbHLH42 | 26146.46 | 9.11 |
| CpbHLH43 | 29541.8 | 6.16 |
| CpbHLH44 | 27474.11 | 5.09 |
| CpbHLH45 | 43581.14 | 5.16 |
| CpbHLH46 | 47071.51 | 6.1 |
| CpbHLH47 | 38605.89 | 9.13 |
| CpbHLH48 | 37078.04 | 4.65 |
| CpbHLH49 | 63252.23 | 8.83 |
| CpbHLH50 | 82270.77 | 5.26 |
| CpbHLH51 | 35786.75 | 6.73 |
| CpbHLH52 | 37593.3 | 4.75 |
| CpbHLH53 | 47880.25 | 7.24 |
| CpbHLH54 | 48664.91 | 8.96 |
| CpbHLH55 | 36256.64 | 5.14 |
| CpbHLH56 | 110499.72 | 4.91 |
| CpbHLH57 | 37360.94 | 5.6 |
| CpbHLH58 | 61481.12 | 6.45 |
| CpbHLH59 | 24377.89 | 6.24 |
| CpbHLH60 | 59286.35 | 6.75 |
| CpbHLH61 | 31386.42 | 5.9 |
| CpbHLH62 | 17842.07 | 6.91 |
| CpbHLH63 | 91846.48 | 5.53 |
| CpbHLH64 | 33397.79 | 5.48 |
| CpbHLH65 | 26775.49 | 7.64 |
| CpbHLH66 | 13632.55 | 9.66 |
| CpbHLH67 | 20657.37 | 6.08 |
| CpbHLH68 | 26397.14 | 8.25 |
| CpbHLH69 | 28974.78 | 6.07 |
| CpbHLH70 | 43928.54 | 7.7 |
| CpbHLH71 | 40377.55 | 8.05 |
| CpbHLH72 | 60767.65 | 6.25 |
| CpbHLH73 | 44767.67 | 5.8 |
| CpbHLH74 | 54221.18 | 6.05 |
| CpbHLH75 | 67940.38 | 6.29 |
| CpbHLH76 | 36693.44 | 6.42 |
| CpbHLH77 | 30912.44 | 8.8 |
| CpbHLH78 | 44991.38 | 5.57 |
| CpbHLH79 | 78524.12 | 5.9 |
| CpbHLH80 | 29753.23 | 5.59 |
| CpbHLH81 | 28184.04 | 5.29 |
| CpbHLH82 | 43121.41 | 4.92 |
| CpbHLH83 | 45842.95 | 6.28 |
| CpbHLH84 | 27373.81 | 9.4 |
| CpbHLH85 | 37210.29 | 4.81 |
| CpbHLH86 | 175494.74 | 7.16 |
| CpbHLH87 | 87545 | 4.78 |
| CpbHLH88 | 42122.43 | 5.26 |
| CpbHLH89 | 79987.57 | 5.54 |
| CpbHLH90 | 36736.34 | 5.38 |
| CpbHLH91 | 35766.63 | 6.26 |
| CpbHLH92 | 36999.91 | 5.08 |
| CpbHLH93 | 40692.85 | 7.15 |
| CpbHLH94 | 43977.06 | 9.09 |
| CpbHLH95 | 87758.71 | 8.87 |
| CpbHLH96 | 37777.32 | 5.31 |
| CpbHLH97 | 29526.02 | 8.54 |
| CpbHLH98 | 48417.23 | 7.05 |
| CpbHLH99 | 28001.92 | 7.07 |
| CpbHLH100 | 24896.06 | 5.92 |
| CpbHLH101 | 38698.85 | 7.7 |
| CpbHLH102 | 55102.71 | 5.49 |
| CpbHLH103 | 48562.53 | 5.45 |
| CpbHLH104 | 46455.86 | 5.88 |
| CpbHLH105 | 29482.97 | 8.91 |
| CpbHLH106 | 39136.53 | 9.02 |
| CpbHLH107 | 40963.04 | 6.03 |
| CpbHLH108 | 29149.03 | 6.56 |
| CpbHLH109 | 27128.86 | 6.71 |
| CpbHLH110 | 20614.3 | 6.11 |
| CpbHLH111 | 28991.95 | 6.24 |
| CpbHLH112 | 17695.96 | 7.74 |
| CpbHLH113 | 45606.54 | 8.3 |
| CpbHLH114 | 56354.24 | 5.44 |
| CpbHLH115 | 56786.36 | 5.18 |
| CpbHLH116 | 60770.74 | 6.26 |
| CpbHLH117 | 52551.34 | 6.94 |
| CpbHLH118 | 47588.78 | 7.69 |
| CpbHLH119 | 39934.06 | 4.93 |
| CpbHLH120 | 42202.05 | 6.51 |
| CpbHLH121 | 47894.76 | 5.62 |
| CpbHLH122 | 34677.68 | 5.52 |
| CpbHLH123 | 65159.06 | 5.38 |
| CpbHLH124 | 60605.61 | 5.92 |
| CpbHLH125 | 37398.31 | 6.11 |
| CpbHLH126 | 10756.94 | 7.98 |
| CpbHLH127 | 26509.55 | 7.75 |
| CpbHLH128 | 54438.5 | 6.01 |
| CpbHLH129 | 39661.07 | 6.73 |
| CpbHLH130 | 38497.46 | 6.27 |
| CpbHLH131 | 61869.78 | 6.91 |
| CpbHLH132 | 51849.19 | 6.65 |
| CpbHLH133 | 47684.97 | 5.89 |
| CpbHLH134 | 27674.1 | 6.45 |
| CpbHLH135 | 55195.29 | 6.73 |
| CpbHLH136 | 28064.15 | 6.32 |
| CpbHLH137 | 37861.56 | 5.58 |
| CpbHLH138 | 39537.5 | 5.92 |
| CpbHLH139 | 57816.55 | 5.36 |
| CpbHLH140 | 38307.11 | 6.19 |
| CpbHLH141 | 38220.03 | 6.19 |
| CpbHLH142 | 10587.97 | 8.77 |
| CpbHLH143 | 34368.71 | 8.28 |
| CpbHLH144 | 16312.45 | 7.79 |
| CpbHLH145 | 21529.95 | 9.62 |
| CpbHLH146 | 74797.92 | 5.28 |
| CpbHLH147 | 34191.03 | 7.73 |
| CpbHLH148 | 54257.9 | 6.4 |
| CpbHLH149 | 55672.55 | 6.26 |
| CpbHLH150 | 82979.14 | 5.52 |
| CpbHLH151 | 31827.48 | 9.38 |
| CpbHLH152 | 25839.35 | 6.64 |
| CpbHLH153 | 55137.42 | 9.31 |
| CpbHLH154 | 50428.21 | 5.65 |
| CpbHLH155 | 27543.43 | 9.45 |
| CpbHLH156 | 23497.65 | 6.61 |
| CpbHLH157 | 22254.78 | 8.55 |
| CpbHLH158 | 38698.85 | 7.7 |
| CpbHLH159 | 32224.62 | 6.34 |

**Supplementary Table S3. Prediction of DNA binding type based on conserved amino acid residues in the basic region.**

| **Gene ID** | **9** | **13** | **16** | **17** | **Type** |
| --- | --- | --- | --- | --- | --- |
| CpbHLH1 | H | E | R | R | G-box |
| CpbHLH2 | H | E | R | R | G-box |
| CpbHLH3 | S | R | T | D | Non-E-box |
| CpbHLH4 | H | E | R | R | G-box |
| CpbHLH5 | H | E | R | R | G-box |
| CpbHLH6 | N | E | R | R | Non-G-box |
| CpbHLH7 | H | E | R | R | G-box |
| CpbHLH8 | H | E | R | R | G-box |
| CpbHLH9 | Q | A | R | R | Non-E-box |
| CpbHLH10 | H | E | R | R | G-box |
| CpbHLH11 | H | E | R | R | G-box |
| CpbHLH12 | H | E | R | R | G-box |
| CpbHLH13 | / | E | R | R | Non-G-box |
| CpbHLH14 | H | A | R | R | Non-E-box |
| CpbHLH15 | K | E | R | R | Non-G-box |
| CpbHLH16 | H | E | R | R | G-box |
| CpbHLH17 | Q | A | R | R | Non-E-box |
| CpbHLH18 | H | E | R | R | G-box |
| CpbHLH19 | H | E | R | R | G-box |
| CpbHLH20 | H | E | R | R | G-box |
| CpbHLH21 | Q | A | R | R | Non-E-box |
| CpbHLH22 | H | E | R | R | G-box |
| CpbHLH23 | N | E | R | R | Non-G-box |
| CpbHLH24 | H | E | R | R | G-box |
| CpbHLH25 | H | E | R | R | G-box |
| CpbHLH26 | H | E | R | R | G-box |
| CpbHLH27 | Q | A | R | R | Non-E-box |
| CpbHLH28 | H | E | R | R | G-box |
| CpbHLH29 | H | E | R | R | G-box |
| CpbHLH30 | N | E | R | R | Non-G-box |
| CpbHLH31 | H | E | R | R | G-box |
| CpbHLH32 | K | E | R | R | Non-G-box |
| CpbHLH33 | H | E | R | R | G-box |
| CpbHLH34 | H | E | R | R | G-box |
| CpbHLH35 | H | E | R | R | G-box |
| CpbHLH36 | H | E | R | R | G-box |
| CpbHLH37 | H | E | R | R | G-box |
| CpbHLH38 | K | E | K | R | Non-E-box |
| CpbHLH39 | S | R | T | D | Non-E-box |
| CpbHLH40 | H | E | R | R | G-box |
| CpbHLH41 | H | E | R | R | G-box |
| CpbHLH42 | N | E | R | R | Non-G-box |
| CpbHLH43 | R | E | R | R | Non-G-box |
| CpbHLH44 | N | E | R | R | Non-G-box |
| CpbHLH45 | Q | A | R | R | Non-E-box |
| CpbHLH46 | H | E | R | R | G-box |
| CpbHLH47 | H | E | R | R | G-box |
| CpbHLH48 | N | E | R | R | Non-G-box |
| CpbHLH49 | H | E | R | R | G-box |
| CpbHLH50 | H | E | R | R | G-box |
| CpbHLH51 | K | E | R | R | Non-G-box |
| CpbHLH52 | N | E | R | R | Non-G-box |
| CpbHLH53 | H | E | R | R | G-box |
| CpbHLH54 | H | E | R | R | G-box |
| CpbHLH55 | H | E | R | R | G-box |
| CpbHLH56 | N | E | R | R | Non-G-box |
| CpbHLH57 | H | E | R | R | G-box |
| CpbHLH58 | H | E | R | R | G-box |
| CpbHLH59 | / | E | R | R | Non-G-box |
| CpbHLH60 | H | E | R | R | G-box |
| CpbHLH61 | H | E | R | R | G-box |
| CpbHLH62 | Q | A | R | R | Non-E-box |
| CpbHLH63 | F | D | R | S | Non-E-box |
| CpbHLH64 | Q | A | R | R | Non-E-box |
| CpbHLH65 | K | E | R | R | Non-G-box |
| CpbHLH66 | H | E | R | R | G-box |
| CpbHLH67 | / | E | R | R | Non-G-box |
| CpbHLH68 | H | E | R | R | G-box |
| CpbHLH69 | H | E | R | R | G-box |
| CpbHLH70 | R | E | R | R | Non-G-box |
| CpbHLH71 | R | E | R | R | Non-G-box |
| CpbHLH72 | H | E | R | R | G-box |
| CpbHLH73 | H | E | R | R | G-box |
| CpbHLH74 | H | E | R | R | G-box |
| CpbHLH75 | H | E | R | R | G-box |
| CpbHLH76 | H | E | R | R | G-box |
| CpbHLH77 | Q | A | R | R | Non-E-box |
| CpbHLH78 | H | E | R | R | G-box |
| CpbHLH79 | H | E | R | R | G-box |
| CpbHLH80 | R | E | R | R | Non-G-box |
| CpbHLH81 | N | E | R | R | Non-G-box |
| CpbHLH82 | Q | A | R | R | Non-E-box |
| CpbHLH83 | H | E | R | R | G-box |
| CpbHLH84 | H | E | R | R | G-box |
| CpbHLH85 | N | E | R | R | Non-G-box |
| CpbHLH86 | H | E | R | R | G-box |
| CpbHLH87 | H | E | R | R | G-box |
| CpbHLH88 | A | D | R | R | Non-E-box |
| CpbHLH89 | H | E | R | R | G-box |
| CpbHLH90 | H | E | R | R | G-box |
| CpbHLH91 | K | E | R | R | Non-G-box |
| CpbHLH92 | N | E | R | R | Non-G-box |
| CpbHLH93 | H | E | R | R | G-box |
| CpbHLH94 | H | E | R | R | G-box |
| CpbHLH95 | H | E | R | R | G-box |
| CpbHLH96 | H | E | R | R | G-box |
| CpbHLH97 | H | E | R | R | G-box |
| CpbHLH98 | Q | A | R | R | Non-E-box |
| CpbHLH99 | H | E | R | R | G-box |
| CpbHLH100 | K | E | R | R | Non-G-box |
| CpbHLH101 | R | E | R | R | Non-G-box |
| CpbHLH102 | H | E | R | R | G-box |
| CpbHLH103 | H | E | R | R | G-box |
| CpbHLH104 | H | E | R | R | G-box |
| CpbHLH105 | Q | A | R | R | Non-E-box |
| CpbHLH106 | R | E | R | R | Non-G-box |
| CpbHLH107 | H | E | R | R | G-box |
| CpbHLH108 | H | E | R | R | G-box |
| CpbHLH109 | H | E | R | R | G-box |
| CpbHLH110 | / | E | R | R | Non-G-box |
| CpbHLH111 | H | E | R | R | G-box |
| CpbHLH112 | Q | A | R | R | Non-E-box |
| CpbHLH113 | R | E | R | R | Non-G-box |
| CpbHLH114 | H | E | R | R | G-box |
| CpbHLH115 | H | E | R | R | G-box |
| CpbHLH116 | H | E | R | R | G-box |
| CpbHLH117 | H | E | R | R | G-box |
| CpbHLH118 | R | E | R | R | Non-G-box |
| CpbHLH119 | Q | A | R | R | Non-E-box |
| CpbHLH120 | H | E | R | R | G-box |
| CpbHLH121 | H | E | R | R | G-box |
| CpbHLH122 | K | E | R | R | Non-G-box |
| CpbHLH123 | N | E | R | R | Non-G-box |
| CpbHLH124 | H | E | R | R | G-box |
| CpbHLH125 | H | E | R | R | G-box |
| CpbHLH126 | T | R | T | D | Non-E-box |
| CpbHLH127 | H | E | R | R | G-box |
| CpbHLH128 | N | E | R | R | Non-G-box |
| CpbHLH129 | H | E | R | R | G-box |
| CpbHLH130 | H | E | R | R | G-box |
| CpbHLH131 | H | E | R | R | G-box |
| CpbHLH132 | H | E | R | R | G-box |
| CpbHLH133 | H | E | R | R | G-box |
| CpbHLH134 | H | E | R | R | G-box |
| CpbHLH135 | H | E | R | R | G-box |
| CpbHLH136 | Q | A | R | R | Non-E-box |
| CpbHLH137 | H | E | R | R | G-box |
| CpbHLH138 | H | E | R | R | G-box |
| CpbHLH139 | N | E | R | R | Non-G-box |
| CpbHLH140 | H | E | R | R | G-box |
| CpbHLH141 | H | E | R | R | G-box |
| CpbHLH142 | S | S | S | D | Non-E-box |
| CpbHLH143 | H | E | R | R | G-box |
| CpbHLH144 | H | E | R | R | G-box |
| CpbHLH145 | / | E | R | R | Non-G-box |
| CpbHLH146 | H | E | R | R | G-box |
| CpbHLH147 | H | E | R | R | G-box |
| CpbHLH148 | H | E | R | R | G-box |
| CpbHLH149 | N | E | R | R | Non-G-box |
| CpbHLH150 | S | E | R | R | Non-G-box |
| CpbHLH151 | H | E | R | R | G-box |
| CpbHLH152 | K | E | R | R | Non-G-box |
| CpbHLH153 | H | E | R | R | Non-G-box |
| CpbHLH154 | Q | A | R | R | Non-E-box |
| CpbHLH155 | H | E | R | R | Non-G-box |
| CpbHLH156 | K | E | R | R | Non-G-box |
| CpbHLH157 | H | E | R | R | Non-G-box |
| CpbHLH158 | R | E | R | R | Non-G-box |
| CpbHLH159 | K | E | R | R | Non-G-box |

**Supplementary Table S4. Twenty different motifs commonly observed in CpbHLH proteins.**

| **Motif** | **Motif Sequence** | **Annotation** |
| --- | --- | --- |
| Motif1 | HSLAERRRREKJNERLKALRSLVPNCSKM | Helix-loop-helix DNA-binding domain |
| Motif2 | DKASMLDEAINYVKELQRQVQELSMKLE | Helix-loop-helix DNA-binding domain |
| Motif3 | IRICCERRPGLLLKJMSALESLGLTILHA | ACT domain |
| Motif4 | DYIHVRARRGQATDS |  |
| Motif5 | YECERAKEARSAGIQTLVCIPTSSGVVELGSTELIREDLGL | bHLH-MYC and R2R3-MYB transcription factors N-terminal |
| Motif6 | NVTTVDEMVLYSLSVKVEEGCKLTSVDEIAAAVHQMLRRI |  |
| Motif7 | IPAAFAAQGQAPGNKLVPFIGYPGVAMWQFMPPAAVDTSQDHVLRPPVA |  |
| Motif8 | JIQSRPEWWVYAIFWQTSKDDNGHVVLSWGDGHFRGTRD | bHLH-MYC and R2R3-MYB transcription factors N-terminal |
| Motif9 | EIKELKQEKNELREEKASLKADKEKLEVQ |  |
| Motif10 | EDLTDSEWFYLVSMSFSFSIGQGLPGRAYANGQPVWLTGAH | bHLH-MYC and R2R3-MYB transcription factors N-terminal |
| Motif11 | SNDGTEHQVAKLMEEDMGAAMQFLQSKALCIMPISLASAIYQTQPSDT |  |
| Motif12 | GGTISISSVYSQGLLNSLTQALQSSGVDLSQASISVQIDLGKQANRGKTG | ACT domain |
| Motif13 | LPVTTSRRKRRRTRSTKNKEEIENQRMT |  |
| Motif14 | SSKSALAEIEVRJIESDAL |  |
| Motif15 | VISCFNDFAMDASCSEDMDZG |  |
| Motif16 | MAAMREMIFRIAAMQPIHIDP |  |
| Motif17 | WWQSAPQPGILEWADGYYNGDIKTRKTVQPIELNADZLGLQRSZQLRELY | bHLH-MYC and R2R3-MYB transcription factors N-terminal |
| Motif18 | TVNPRLDFNIESLLSKDIFPSRGATFPTJ |  |
| Motif19 | NRPHALEHHLSLPKTSLEMATAEKFJQFQ |  |
| Motif20 | DGISSDNGHQNAMHSYATNSFGMDSWDNTNSIIFSAPPGERAKNADNDIF |  |
